# Mechanisms of leukemogenesis induced by bovine leukemia virus: prospects for novel anti-retroviral therapies in human

**DOI:** 10.1186/1742-4690-4-18

**Published:** 2007-03-16

**Authors:** Nicolas Gillet, Arnaud Florins, Mathieu Boxus, Catherine Burteau, Annamaria Nigro, Fabian Vandermeers, Hervé Balon, Amel-Baya Bouzar, Julien Defoiche, Arsène Burny, Michal Reichert, Richard Kettmann, Luc Willems

**Affiliations:** 1 Molecular and Cellular Biology, Faculté Universitaire des Sciences Agronomiques, Gembloux, Belgium; 2 National Veterinary Research Institute, Pulawy, Poland; 3 Luc Willems, National fund for Scientific Research, Molecular and Cellular Biology laboratory, 13 avenue Maréchal Juin, 5030 Gembloux, Belgium

## Abstract

In 1871, the observation of yellowish nodules in the enlarged spleen of a cow was considered to be the first reported case of bovine leukemia. The etiological agent of this lymphoproliferative disease, bovine leukemia virus (BLV), belongs to the deltaretrovirus genus which also includes the related human T-lymphotropic virus type 1 (HTLV-1). This review summarizes current knowledge of this viral system, which is important as a model for leukemogenesis. Recently, the BLV model has also cast light onto novel prospects for therapies of HTLV induced diseases, for which no satisfactory treatment exists so far.

## 1. Background

The occurrence in cattle of a disease called "leukosis" was first reported by Leisering who described as early as in 1871 the presence of yellowish nodules in the enlarged spleen of a cow [1]. In fact, spleen disruption consecutive to tumor formation is one of the most spectacular clinical manifestations of bovine leukemia. These tumors which result from a local accumulation of transformed B cells also infiltrate other tissues such as liver, heart, eye, skin, lung and lymph nodes (reviewed in [2-5]). Two types of bovine leukemia can be dissociated on the basis of their epidemiology: Enzootic Bovine Leukosis (EBL), a disease caused by a retrovirus called BLV (Bovine Leukemia Virus), and sporadic bovine leukosis which is not transmissible. Besides the lethal form of BLV-induced leukemia, persistent lymphocytosis (PL) is characterized by a permanent and relatively stable increase in the number of B lymphocytes in the peripheral blood. The PL stage, which affects approximately one third of infected animals, is considered to be a benign form of the disease resulting from the accumulation of untransformed B lymphocytes. Finally, viral infection is asymptomatic in the majority of BLV-infected animals; in these settings, fewer than 1 % of peripheral blood cells in animals are found to be infected by virus.

BLV is transmitted horizontally through the transfer of infected cells via direct contact, through milk and possibly by insect bites [6]. However, iatrogenic procedures like dehorning, ear tattooing and, any use of infected needles contribute significantly to viral spread [7-10]. BLV is nowadays highly prevalent in several regions of the world (e.g. United States) and induces major economical losses in cattle production and export [11-21]. For instance, the loss to the dairy industry due to BLV in 2003 was estimated annually at $525 million. In contrast, Denmark was the first country where the virus has been eradicated through the systematic destruction of infected herds. It is remarkable that the identification of infected animals was performed on basis of peripheral blood cell counts without the availability of specific serological tests (Bendixen's key) [22]. BLV is now almost completely eradicated from the European Union after many years of culling infected animals. Since these costly eradication programs are only possible in regions where viral prevalence is low, other strategies have also been considered including isolation of infected animals, passive immunization with colostrum, vaccination with viral proteins or attenuated strains, as well as some other exotic approaches ([5,23-34] and references therein). None of these latter methods currently achieve the optimal combination of efficiency, economy and safety.

Domestic cattle are the natural hosts for BLV. The existence of wild reservoirs remains controversial, but convincing evidence indicates that BLV indeed persists in water buffaloes [35-37]. Experimental transmissions of BLV have been reported in many species including rabbits [38-40], rats [41,42], chickens [43], pigs [44], goats [45] and sheep [9,46-48]. However, only sheep consistently develop leukemia whereas rabbits present immune dysfunctions (but no tumors, in a finding different from rabbits inoculated with HTLV [49]). Rare cases of experimental transformation were reported in goats, rats and even chicken. Despite successful infection of a series of cell lines *in vitro *[50-53], BLV does not persistently infects cat, dog, monkey or human although viral-specific seroconversion might occur in these species. Epidemiological studies have shown that consumption of raw milk from BLV-infected cattle does not increase the frequency of leukemia in man (reviewed in [54-56]). Therefore, it is unlikely that BLV infects, replicates and induces cancer in humans, although this cannot be formally excluded [57]. Instead, four BLV related retroviruses have been isolated in man: Human T-lymphotropic viruses type 1 to 4 (HTLV-1 to -4) [58-60]. Among these, HTLV-1 infects about 20 million people worldwide, a fraction of whom (about 2–3 %) progress to develop acute T-cell leukemia (ATL) or HTLV-Associated Myelopathy/Tropical Spastic Paraparesis (HAM/TSP), a neuroinflammatory disease of the central nervous system.

## 2. The BLV genome

In addition to the structural *gag*, *pol *and *env *genes required for the synthesis of the viral particle, the BLV genome contains a X region located between the envelope and the 3' long terminal repeat [61-63], as also observed in other deltaretroviruses [58,60]. Phylogenetic comparisons of different strains, using the *pol *gene as a reference, indicate that BLV and primate T-lymphotropic viruses (PTLV) sequences differ by 42 % [64]; thus BLV forms a distinct clade amongst retroviruses. Within the BLV subgroup, the sequence divergence was below 6% in *pol *and *env *indicating a high degree of conservation among different geographical strains [24,25,65-67]. Although the reasons are unknown, this genomic stability might result from a higher fidelity of reverse transcription or from strict replication constraints.

### The genomic RNA

**Figure 1:**
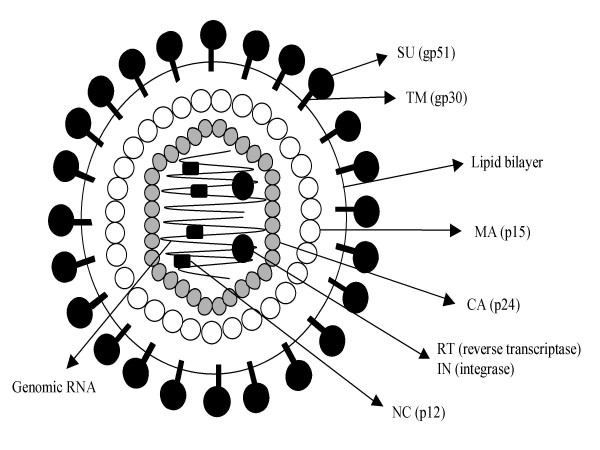
**Schematic representation of the BLV viral particle**. Two copies of single stranded genomic RNA are packaged in a viral particle. The CA (p24) proteins form a capsid that contains the viral RNA in interaction with nucleocapsid NC (p12). Two enzymatic proteins (RT and IN) required, respectively, for reverse transcription and integration of the viral genome are also packaged into the capsid. The matrix protein MA (p15) interconnects the capsid and the outer envelope that is formed by a lipid bilayer of cellular origin in which a complex of viral proteins (gp51 SU and gp30 TM) are inserted.

Morphologically, the viral particle with a diameter ranging between 60 and 125 nm, is constituted by a central electron dense nucleoid surrounded by an outer viral envelope [68,69] (Figure 1). Infectious virions contain 60–70 S ribonucleic acids resulting from the association of two 38 S poly-A containing RNA molecules [70].

**Figure 2:**
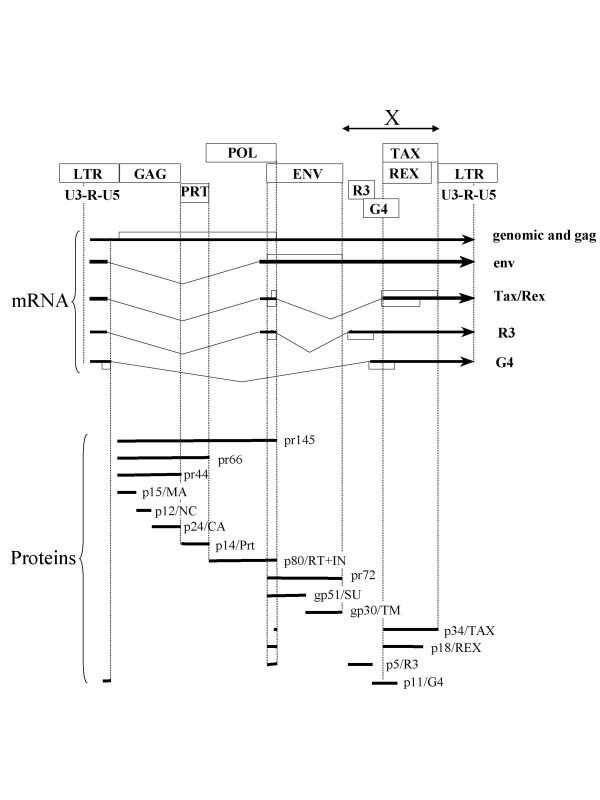
**Structure of the BLV provirus: genes, RNA transcripts and viral protein**. The provirus is flanked by two identical long terminal repeat sequences (LTRs) and contains the open reading frames (*orfs*) corresponding to gag, prt (protease), pol and env. Several *orfs *coding for Tax, Rex, R3 and G4 are present in the X region between env and the 3'LTR. The genomic RNA transcript initiates and terminates in the 5' and 3' LTRs, respectively. This genomic RNA serves as a template for the expression of the gag-prt-pol precursors (pr145, pr66 and pr44) that are processed in structure and enzymatic proteins: matrix (MA) p15, capsid (CA) p24, nucleocapsid (NC) p12, protease (PRT) p14 and, p80 (RT/IN) harboring reverse transcriptase, RNAse H and integrase activities. A large intron corresponding to gag-prt-pol is excised to yield the envelope (env) RNA. After translation, the pr72 precursor is cleaved in two subunits: the extracellular (SU) gp51 and the transmembrane (TM) gp30 glycoproteins. To generate the Tax/Rex messenger RNA, a second intron is cleaved. This double-spliced RNA encodes both the p34 Tax protein using an initiation codon at the end of pol and Rex which shares the same AUG as Env pr72. Two minor RNAs identified by RT/PCR code for p5 (R3) and p11 (G4). The R3 RNA is similar to the double-spliced Tax/Rex message but the second intron is shorter and splicing occurs at the 5' end of the R3 frame. The R3 protein shares its aminoterminal end with Rex and pr72. In the G4 message, a very large intron is excised between a particular splice donor site different from that of the other viral RNAs and an acceptor just 5' to the G4 frame. The G4 protein initiates at a suboptimal CUG codon located in R of the 5'LTR.

Transcription of the genomic RNA initiates at the U3/R boundary of the 5' LTR (long terminal repeat) and terminates at the polyadenylation site (Figure 2). This genomic RNA interacts with matrix (MA) p15 and nucleocapsid (NC) p12 proteins and dimerizes through a region surrounding the primer binding site [71,72]. Efficient encapsidation of the RNA requires two regions: a primary signal which is located in the untranslated leader region between the primer binding site and near the *gag *start codon and a second fragment within the 5' end of the *gag *gene [73]. Both regions fold into secondary structures that are required for efficient packaging [74-76]. The primary encapsidation signal does not overlap with a structure important for cell-free dimer formation but fits with a region interacting with MA. Replacement of the BLV RNA region containing the primary and secondary encapsidation signals with a similar region from HTLV-1 or -2 yields a recombinant virus competent for replication in cell culture. Heterologous RNAs can be packaged into BLV particles by means of a minimal RNA packaging sequence [77]. Viral RNA packaging requires the involvement of both the MA and NC domains of Pr145^gag-pol^, in particular conserved basic residues of MA as well as residues of the zinc finger domains of NC [78].

The 3' end of the genomic RNA also contains a highly structured region (RxRE), which is needed to mediate RNA transport from the nucleus to the cytoplasm (see paragraph on post-transcriptional regulation by Rex). After transcription and nuclear export, the genomic RNA can either be directly translated to yield the Pr145^gag-pol ^precursor or incorporated into a budding viral particle.

### The long terminal repeat

The genomic RNA is a 9 kb ribonucleic acid molecule flanked by R regions of the long terminal repeats (Figure 2). In addition to this transcript, a series of other RNAs can be synthesized in infected cells with two major species of 5.1 kb (the *env *RNA) and 2.1 kb (*tax/rex*) and several less abundant RNAs coding for R3 and G4 [79,80]. All these transcripts initiate at the boundary between U3 and R (the CAP site) and terminate with polyadelylation at the end of R in the 3' LTR. The U3 region contains the canonical promoter "CAT" box (CCAACT at coordinates -97 to -92) and "TATA" boxes (GATAAAT between -44 and -38). Another series of sites mainly located in the U3 region of the 5' LTR regulate viral transcription [81,82].

A key regulatory element of the LTR is a triplicate copy of a 21 bp sequence called TxRE harboring in the middle of the sequence an almost perfectly conserved cyclic-AMP responsive element (CRE) with an overlapping E box motif. Only two of these TxREs are required for infectivity and pathogenicity in sheep [83]. In gel retardation assays with primary B lymphocyte lysates, the TxRE element interacts with the CREB, ATF-1 and ATF-2 transcription factors and the amount of protein-DNA complex correlates with the level of viral expression [84,85]. The CREB/ATF transcription factors regulate LTR-directed transcription when activated by two cellular protein kinases (i.e. PKA and CaMKIV). The 21 bp enhancer is also a target of the Tax protein, a viral transcriptional activator which increases the binding of CREB to DNA [86]. In fact, the internal CRE-like sequences (AGACGTCA, TGACG GCA, TGACCTCA), a property which is shared by the related HTLV-1 LTR [87], are close to but different from the consensus "TGACGTCA". Restoring a perfect CRE sequence into the 21 bp sequences increases the BLV LTR promoter activity in reporter assays, but interferes with viral replication *in vivo *[88]. Indeed, the proviral loads are drastically reduced in sheep infected with a virus harboring perfect consensus CRE elements (see section 4, below). Another regulatory process is exerted by E-box motifs which overlap the CRE elements and repress basal LTR-driven gene expression [89].

Although the 21 bp enhancer is a major regulator of viral expression, other U3 elements also modulate LTR-directed transcription. Among them, a NF-κB-related site located between the proximal and middle 21 bp enhancers, binds *in vitro *to several members of the kappa B family of proteins including p49, p50 and p65 and confers further transcriptional activation [90,91]. Another motif, located just 5' to the proximal 21 bp, is required for responsiveness of the LTR promoter to glucocorticoids [92,93]. A PUbox located at coordinates -95/-84 bp specifically interacts with PU.1 and the related Ets transcription factor Spi-B [94]. Mutations within this element decrease LTR-driven basal gene expression but does not impair responsiveness to Tax. An E-box motif (5'-CACGTG-3') located downstream of the transcription start site binds the basic helix-loop-helix transcription factors USF1 and USF2 and regulates the LTR promoter [95]. In addition to these U3 elements, viral expression is regulated also by sequences in the R region [81]. Finally, an interferon regulatory factor binding site in U5 interacts with IRF-1 and IRF-2 and stimulates basal expression in the absence of Tax [96]. Viral transcription thus appears to be regulated by numerous sites distributed throughout the 5' LTR.

Viral transcription is regulated at a separate level by epigenetic modifications such as acetylation of histone molecules and DNA methylation. Indeed, cultivation of peripheral blood mononuclear cells (PBMCs) from BLV-infected animals in the presence of histone deacetylase (HDAC) inhibitors significantly increases viral expression [97]. A close correlation links the level of histone acetylation and transcriptional activation of the LTR [89]. HDAC inhibitors synergistically enhance transactivation of the LTR by Tax in a CRE-dependent manner [98]. Trichostatin A increases the occupancy of the CRE elements by CREB/ATF as shown by chromatin immunoprecipitation assays.

DNA methylation could be another means for regulating LTR-transcription. Indeed, *in vitro *methylation of the LTR by CpG methyltransferase SssI reduces LTR activity in luciferase reporter assays [99]. However, only minimal modifications of CpG methylation were detected at all stages examined in BLV-infected cattle and sheep. Further experiments are therefore required to clarify the role of methylation in LTR activity, as has been described in the HTLV system [100].

Finally, viral expression is also regulated at the post-transcriptional level by a viral protein called Rex which interacts with RNA sequences in the 3'LTR located between the AATAAA signal and the polyadenylation site [101]. This region is able to fold into a stable RNA hairpin structure and brings the two transcription termination signals together. Rex binding is required for the nuclear to cytoplasmic export of unspliced and singly spliced, but not for multiply spliced, BLV transcripts.

### The gag and protease genes

The *gag *gene codes for the Pr44^gag ^precursor, a polypeptide subsequently cleaved into the major non-glycosylated proteins of the viral particle (p15, p24 and p12) (Figure 2) [70,102-106]. The matrix (MA) protein p15 (109 aa) which corresponds to the NH_2_-terminal end of the *gag *precursor is a myristylated and phosphorylated polypeptide. MA proteins bind the genomic viral RNA but also interact with the lipid bilayer of the viral membrane. Structurally, MA contains four principal helices that are joined by short loops [107]. The matrix protein can be further proteolytically processed to generate three fragments: p10, a seven amino acids product, and p4 [71]. p10, which is also myristylated, results from the amino-terminal cleavage of MA.

The p12 nucleocapsid (NC) is a proline-rich 69 aa protein that is tightly bound to the packaged genomic RNA [71,108]. In the presence of Zn^2+^, NC interacts with single-stranded nucleic acids with an affinity in the nanomolar range [109].

p24, a neutral and moderately hydrophobic protein, is the major constituent of the capsid (CA) of BLV virions. The CA protein appears to be a major target for the host immune response with high antibody titers found in the sera of infected animals and two defined regions of p24 being recognized by specific T lymphocytes [110,111]. One of the T-cell epitopes overlaps with a domain highly conserved among retroviruses, the major homology region (MHR), which is required for viral infectivity *in vivo *[112]. Based on the use of a monoclonal antibody directed against BLV p24, a common epitope was found to be shared with HTLV CA [113,114]. Interestingly, this cross-reactivity between the capsid antigens of BLV and HTLV-1 suggests an evolutionary relationship between the two viruses. Of note, an immunological cross-reaction is also observed between the BLV and the feline leukemia virus (FeLV) nucleocapsid NC proteins [115].

Different BLV Gag proteins (MA, CA and NC) are derived from the proteolytic cleavage of the Pr44^gag ^precursor. This post-translational maturation is carried out by the viral protease p14 which is encoded by a region located between the *gag *and the *pol *genes. p14 is synthesized from a gag-protease precursor (pr66^gag-prt^) via a frameshift suppression of the gag termination codon by a lysine-specific tRNA [116]. The pr66^gag-prt ^precursor localizes at the surface of polarized cells [117]. The p14 protein, which assembles into dimers, belongs to the aspartyl proteinase group and can be inhibited by pepstatin A [118]. Despite their evolutionary relationship, the BLV and HTLV proteases harbor marked specificities in cleavage site recognition [119].

Overexpression of the Gag polyprotein in mammalian cells generates virus-like particles (VLPs). VLPs production depends on the PPPY motif located in the MA domain and on the amino-terminal glycine involved in Gag myristylation. The PPPY sequence functions as a late domain and plays a role in budding of the viral particle [120,121]

### The pol gene

The *pol *gene is translated via a frameshift mechanism, as a 145-kDa precursor (852 aa) [116]. Pr145 contains all of the tryptic peptides of the gag-protease precursor and thus represents an elongation product of pr66^gag-prt^. The *pol *gene encodes a 80 kDa reverse transcriptase (RT), a RNA-dependent DNA polymerase which is preferentially active in the presence of Mg^2+ ^[122,123]. In fact, the enzyme shows a preference for Mg^2+ ^over Mn^2+ ^in both its DNA polymerase and RNase H activities [124]. BLV RT is relatively resistant to nucleoside triphosphate analogues known to be potent inhibitors of human immunodeficiency virus (HIV-1) reverse transcriptase. Bacterially produced BLV RT is enzymatically active as a monomer even after binding a DNA substrate [125]. Amazingly, sera from some leukemic cattle contain antibodies that inhibit reverse transcriptase activity *in vitro*.

The synthesis of the minus strand DNA by RT begins at the primer binding site for tRNA pro in the genomic template RNA located just 2 bp downstream of U5. BLV reverse transcriptase exhibits a higher fidelity than that from spleen necrosis virus: only 1.2 × 10^-5 ^base mutations (versus 4.8 × 10^-6 ^for SNV) occur per round of replication [126]. In fact, BLV RT shows a fidelity of misinsertion better than that of HIV-1 RT but a similar degree of mispair elongation (i.e. the ability to extend these 3' end mispairs) [124].

After infection of permissive cells, two species of covalently closed circular DNA molecules, harboring one or two LTR copies, are synthesized after reverse transcription [127,128]. Unintegrated viral DNA molecules are abundant in asymptomatic and PL cattle but they appear to be absent during the tumor phase [129]. Insertion of the double-stranded DNA, also known as the provirus, is directed by the virally encoded integrase IN [130,131]. During DNA rearrangement, the integrase recognition sequence includes the 3' end of the U5 LTR region [131]. Once inserted at random sites into the host genome, the provirus is flanked by direct repeats of cellular DNA [132].

### The envelope gene

The sequences coding for the BLV envelope partially overlap in a different frame the 3' end of *pol *by 51 nucleotides [62,133,134]. The envelope gene is transcribed as a 5.1 kb mRNA coding for the pr72^env ^precursor [70,80,103,105,106,135]. The BLV and HTLV envelopes show 36 % of identities in their amino acid sequence. The BLV pr72^env ^precursor is cleaved into two subunits by subtilisin/kesin-like convertases such as furin [136]. The resulting products, the extracellular gp51 (SU) and the transmembrane gp30 (TM) proteins are glycosylated polypeptides [137-140]. SU and TM associate through disulfide bonds, conferring a relatively stable complex [141].

Interestingly, the pr72^env ^precursor polyprotein is not evenly distributed but concentrates predominantly in only one daughter cell [117]. This mechanism might account for the absence of viral antigens in a proportion of the cell progeny and permit persistence of the virus (see hypothetical model in section 6, below)

In contrast to TM which is very poorly immunogenic, the extracellular SU induces massive expression of specific antibodies in infected animals, a property useful for diagnostics and vaccine development. Some monoclonal antibodies (F, G and H) directed towards conformational epitopes of SU exhibit neutralizing activities [137,142,143]. None of the known viral strains are simultaneously lacking F, G and H reactivities, suggesting that loss of these three epitopes probably means loss of infectivity. In addition, rabbit antisera raised against peptides 39–48, 78–92, 144–157 and 177–192 neutralize VSV/BLV pseudotypes *in vitro*, indicating that these epitopes are also implicated in viral infectivity [144,145]. Interestingly, residues 144 to 157 of SU correspond to the region in the HTLV-1 envelope glycoprotein which is also involved in neutralization. Cell fusion, i.e. syncytium formation, is inhibited by sera directed towards peptides 64–73, 98–117 and 177–192. This last sequence (in particular residues P177 and D178 of SU), which stimulates proliferation of lymphocytes isolated from infected cows, is a T-helper epitope. Finally, CD8-dependent cytotoxic activity is associated with peptides 121–140, 131–150 [146], or 24–31 [147,148].

*In silico *modeling indicates that SU glycoprotein oligomerizes as a trimer, in which the putative receptor binding domain (RBD) corresponds to the most efficient neutralizing epitopes [141,143,149]. It should be mentioned here that this cellular receptor for BLV is still unknown, in contrast to those of HTLV-1 (i.e. glut-1 and neuropilin-1) [150,151]. Although a candidate molecule able to interact with SU has been identified [152], it later appeared that this protein corresponds to the δ subunit of adaptor-related protein complex AP3 involved in intracellular vesicle transport [153].

Since cell-free infection by BLV appears to be very inefficient most probably due to virion instability, the main route of transmission is thought to occur through fusion between an infected cell harboring envelope proteins at its surface and a new target lymphocyte [154,155]. The TM transmembrane protein is a key factor during this process which uses a fusion peptide that is able to destabilize the cell membrane through its oblique insertion into the lipid bilayer [156] triggered by the dynamic structural reorganization of the TM aminoterminal end. Two domains of SU, peptide 19–27 which adopts an amphiphilic structure [157] and region 39–103 [136], are also required for efficient cell fusion. Finally, a region of SU localized between residues 104–123 interacts with zinc and affects viral fusion as well as infectivity *in vivo *[158].

In addition to its role in cell fusion, the TM protein is involved in signal transduction via immunoreceptor tyrosine-based activation (ITAM) motifs present in the cytoplasmic tail [159,160]. The critical site of the ITAMs consists of a YXXL sequence (where X represents a variable residue). Similar ITAM motifs are also found in Ig alpha protein of the B cell antigen receptor complex and can be recognized by SH2 domains of signaling proteins. When fused to the CD8 molecule, the TM ITAM motifs are able to transduce signals through the cell membrane after stimulation with an anti-CD8 antibody. These motifs are also important for incorporation of envelope proteins into the virion [161] and are required for infectivity *in vivo *[162].

Using a similar approach of chimeric proteins, the TM cytoplasmic domain has been found to be involved in the modulation of intracellular envelope trafficking [163]. Replacement of two proximal dileucine motifs with alanines increases the surface display of CD8-TM chimeric proteins indicating that these motifs downmodulate cell surface expression of envelope proteins [164].

Besides ITAMs and dileucine motifs, the TM cytoplasmic tail has homology with immunoreceptor tyrosine-based inhibition motifs (ITIMs), which are homologous to B-cell receptor (BCR) and inhibitory co-receptor motifs; however, the functional relevance of these sites remains unclear [165]. The TM cytoplasmic tail also contains typical proline-rich motifs (PXXP) which correspond to SH3 recognition sites. These motifs are not required for viral infectivity but are important for the maintenance of high viral load *in vivo *[166].

Finally, BLV TM interacts with phosphatase SHP-1 that associates with FcγRIIB and acts as a critical negative regulator of BCR signaling [167]. This association suggests the hypothesis that TM may act as a decoy to sequester SHP-1, resulting in up-regulation of BCR signaling.

### The R3 and G4 open reading frames

**Figure 3:**
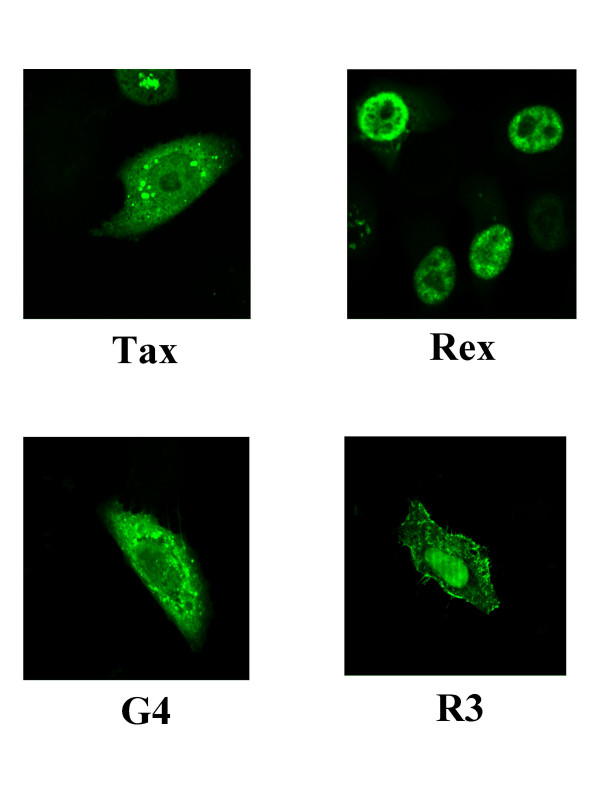
**Subcellular localisation of the Tax, Rex, R3 and G4 proteins**. Hela cells were transfected with expression vectors for Tax, Rex, R3 and G4, cultivated during 24 hours, indirectly marked with FITC-conjugated antibodies and visualized under a fluorescent microscope.

The *R3 *and *G4 *open reading frames (*orfs*) belong to an intermediate region located between the envelope and the *tax/rex *genes. These sequences are transcribed into mRNAs which are present at very low levels *in vivo *[79,168]. The *R3 *mRNA is bicistronic: the first two exons are common to the *tax/rex *messenger but the second intron is shorter and splicing occurs in the middle of the *R3 orf *(Figure 2). The *G4 *mRNA contains only one intron located between an unusual splice donor site at position 502 (instead of 305 for the other viral mRNAs) and an acceptor at the 5' end of G4 (position 7066 according to [62]). *In vitro *translation of these mRNAs yield proteins of 5.5 kDa and 11.6 kDa for R3 and G4, respectively. Initiation of G4 translation occurs at a suboptimal CUG codon in the R region whereas R3 shares the AUG of both the Rex protein and the envelope pr72^env ^precursor. R3 thus contains 17 aminoterminal residues which are also present in Rex and 27 amino acids from the *R3 orf *[79]. Since these proteins share the nucleolus-targeting signal and RNA-binding motifs, R3 could like Rex, be involved in post-transcriptional regulation of viral expression. R3 is located in the nucleus and in cellular membranes (Figure 3), as previously reported for HTLV-1 p12^I^. In contrast, G4, like p13^II^, is localized both in the nucleus and in mitochondria [169].

G4 is likely implicated in cell transformation because its ectopic expression in primary rat embryo fibroblasts induces their immortalization [170]. Furthermore, G4 interacts with farnesyl pyrophosphate synthetase (FPPS), a protein involved in the mevalonate/squalene pathway and in synthesis of FPP, a substrate required for prenylation of Ras [171]. HTLV-1 p13^II ^also specifically interacts with FPPS and colocalizes with G4 in mitochondria, indicating that both accessory proteins exert related functions.

*R3 *and *G4 *are dispensable for infectivity *in vivo *but the integrity of these genes is essential to allow efficient propagation inside the host [83,170,172]. Furthermore, recombinant viruses deleted in *R3 *and *G4 *are very poorly pathogenic in sheep with a single exception out of 20 infected animals having been observed after more than 7 years of latency (Florins et al, manuscript in preparation).

### Rex

Almost 3 decades ago, a 18 kDa protein was identified by *in vitro *translation of RNA isolated from virions [70]. This 18 kDa product was antigenically unrelated to the viral structural proteins and originated from the 3' end of the provirus. Later, it was discovered that this viral protein resulted from the translation of the *rex orf *[173,174]. The *rex *sequences are well conserved amongst various BLV isolates with less than 5% variation [175].

The Rex protein has a punctate nuclear localization, associates with nuclear pores and harbors a nuclear export signal (Figure 3) [24,25,176]. Rex contains a central leucine-rich activation domain and amino-terminal arginine-rich motifs required for RNA-binding and nuclear localization.

Rex is required for the accumulation of genomic and singly-spliced *env *RNAs [101]. This trans-acting regulation of viral mRNA processing requires a 250-nucleotide region located between the AATAAA signal and the polyadenylation site in the 3'LTR. The Rex proteins of HTLV-1 and BLV are interchangeable for purposes of post-transcriptional regulation [177].

The messenger RNA coding for BLV Rex results from the excision of two introns: one is located between the major splice site at nucleotide 305 (according to [62]) and an acceptor at the end of the *pol *gene (position 4649), and the other spans residues 4871 to 7247. This complex double-splicing mechanism yields a 2.1 kb molecule coding not only for Rex, but also for the Tax protein [80,178]. *In vitro*, the *tax/rex *message which is not itself regulated by Rex [101], is present in the cytoplasm during an early phase preceding the accumulation of the other mRNA coding for the structural proteins [179]. Finally, the expression of the *tax/rex *mRNA but not other viral RNAs, is maintained *in vivo *at late phases of leukemia or lymphosarcoma [180].

### The Tax transactivator

The other protein encoded by the 2.1 kb multiply-spliced mRNA is Tax, a transcriptional activator of viral expression. Initiation of *tax *translation occurs at a methionine residue located just upstream of the *pol *stop codon [80,181]. The Tax *orf *is the largest of the X region located between the *env *gene and the 3' LTR (Figure 2). The Tax protein is a target of the host immune response with T and B epitopes corresponding to regions 110–130/131–150 and 261–280, respectively [182].

The importance of *tax *has been suggested by the absence of deletions affecting this *orf *during the process of leukemogenesis [183,184]. However, some proviruses harboring deletions in the central portion of the genome do not contain the second exon required for initiation of Tax. These deletants are thus unable to express Tax, although all of them could at least in theory, express G4. It is still possible that a Tax protein is synthesized via other splicing processes or is provided in *trans *by other infected cells [185]. Besides these speculations, it is clear that the integrity of the *tax *gene is essential for viral infectivity *in vivo *[83,186].

The Tax protein is rich in proline (13 %) and leucine (16 %) residues and has a relatively short half life (5 to 6 hours) [178]. It is mainly localized in the nucleus, although significant amounts are also present in the cytosol [187,188] (Figure 3). Tax is post-translationally modified by phosphorylation of two serine residues (106 and 293) and exhibits at least three forms with measured isoelectric points of 6.05, 6.25 and 6.45 [188,188,189]. Although its calculated molecular weight is 34,328, Tax migrates as a 34–38 kDa product, probably due to its phosphorylation.

The first identified function of Tax is activation of viral transcription [190,191]. This mechanism of transactivation by Tax requires interaction with cellular transcription factors, like CREB, which bind to the 21 bp enhancer elements in the 5'LTR. A very narrow range of variations is compatible with full transactivation activity, suggesting that the present molecular structure of Tax results from heavy evolutionary constraints [192,193,175]. In addition to the main phosphorylation sites at serines 106 and 293, Tax is structurally characterized by the presence of an aminoterminal zinc finger and by a leucine-rich activation domain located between residues 157 and 197 [188,194]. Deletion of the activation domain or substitutions of amino acids involved in the zinc finger completely abolish Tax's transactivation activity. The region between amino acids 245 and 265 of the Tax protein reduces LTR-directed transactivation [195]. A Tax mutant within this region, which also harbors increased c-fos transactivation activity [196], does not propagate virus at higher rates *in vivo *[197]. PBMCs infected with the Tax mutant virus are less prone to undergo intrinsic apoptosis *ex vivo*, a process which involves the Bcl-xl protein [198].

Besides its function as a transcriptional activator, Tax induces immortalization of primary rat embryo fibroblasts (REF) [170,194,199]. In addition, Tax cooperates with the Ha-ras oncogene to induce full transformation of cells that form tumors when injected into nude mice, a property shared with G4 (see above). These activities which are also seen with the Myc oncogene, underline the immortalizing potential of Tax. Tax is thus not strictly an oncogene because it does not have a cellular counterpart but behaves as such in a way similar to the well defined Myc protein. The oncogenic potential of Tax can be dissociated from its transcriptional activation potential by specific mutations. Alterations of the zinc finger yield transactivation-deficient but transformation-competent mutants [194]. In contrast, the main phosphorylation sites are dispensable for transactivation but are required for oncogenicity in the REF system (see section 4) [200,201].

The mechanisms by which Tax induces transformation are still largely unknown. Expression of Tax in primary ovine B lymphocytes, which are dependent on CD154 and interleukin-4, impacts cell proliferation and survival leading to cytokine independent growth [202]. This immortalization process correlates with increased Bcl-2 protein levels, nuclear accumulation of NFκB and a series of intracellular pathways which remain to be characterized [203]. Tax also inhibits base-excision DNA repair of oxidative damage, potentially increasing the accumulation of ambient mutations in cellular DNA [204].

To further understand its mechanisms of action, Tax-associated cellular interacting proteins have been identified using a two-hybrid approach. For example, Tax directly binds to tristetraprolin (TTP), a post-transcriptional modulator of TNFα expression [187]. Tax promotes nuclear accumulation of TTP and restores TNFα expression by inhibiting TTP. Interestingly, this process is shared by the HTLV-1 Tax protein, supporting a key role of this process during cell transformation. Another Tax-interacting protein is MSX2, a general repressor of gene expression, including LTR-dependent transcription [205]. MSX2 repression can be counteracted by overexpression of the CREB2 and RAP74 transcription factors. A third Tax-binding protein is the G protein β subunit [206]. In conditional Tax-1-expressing transformed T-lymphocytes, Tax expression correlates with activation of the SDF-1/CXCR4 pathway.

## 3. BLV infects B lymphocytes

Although it has been reported that BLV could persist in other cell types [207-214], it seems clear that the major target of the virus is a B lymphocyte which expresses surface immunoglobulin M [215-219]. In addition to B lymphocytes, BLV also persists in cells of the monocyte/macrophage lineage. Immunoglobulin γ heavy chains are frequently found on lymphoma cells from cattle, consistently with a mature B cell phenotype [219,220]. Sequencing of VDJ rearrangements in IgM-secreting B lymphocytes from a BLV-infected cow indicates that IgM antibodies are functional, exhibit polyspecific reactivity and contain exceptionally long CDR3H [221]. Such long HCDR3s, which are also often found in poor outcome chronic lymphocytic leukemia patients (B-CLL), characterize antibodies directed towards negatively charged autoantigens (e.g., DNA) [222].

In addition to these markers pertaining to B lymphocytes, infected cells frequently co-express the CD5 molecule. B cell lymphocytosis essentially results from an increased proliferation of circulating CD5+ B lymphocytes associated with a lower but significant increase of the CD5- B cell population [223-226]. Although the provirus has been detected in both CD5+ and CD5- B lymphocytes from infected animals, lymphosarcoma cells appear to exhibit mainly, but not exclusively, the CD5+ B phenotype [220]. CD5 physically associates with the BCR in B lymphocytes from normal but not from PL cattle [227]. BCR crosslinking decreases apoptosis of CD5+ B cells from uninfected animals but does not impact on those of PL cattle in which CD5 is already dissociated from the BCR. In contrast to CD5-negative B cells, BCR in B cells of PL cattle resists movement into lipid rafts upon stimulation and is only weakly internalized [228]. Disruption of CD5-BCR interactions and subsequent decreased apoptosis in antigenically stimulated B cells may thus be a mechanism of BLV-induced PL.

In contrast, the CD5 molecule is often not expressed on tumor cells from BLV-infected sheep [229,230]. Favored growth of CD5 positive cells might result from a difference in susceptibility to apoptosis [231]. Another marker, the CD11b integrin molecule better defines the leukemic cell populations, although the virus infects both CD11b+ and CD11b- cells. These two populations also exhibit marked differences in cell kinetics (see section 6). In addition, the BLV-target cells express the IL-2 receptor (CD25) and the major histocompatibility class II complex or a related molecule previously called the tumor associated antigen (TAA) [219,220,232-234]. This antigen is probably the most frequently expressed protein at the surface of BLV target cells. Monoclonal antibodies recognizing this molecule inhibit the growth of BLV-infected lymphoid cells and induce tumor regression. Molecular cloning of cDNAs corresponding to bovine MHC II (BoLA) indicated that the TAA is closely related, but not identical to BoLA-DR. Of note, B lymphocytes from PL cows express increased spontaneous expression of the MHC class II molecule [235].

To summarize, it appears that the target cell for BLV is MHCII+ surface IgM+, CD5+ and CD11b+ with some fluctuations for the three latter markers at late stages of the disease. In contrast, HTLV-1 clearly infects other cell types CD4+ and CD8+ T lymphocytes [236,237], underscoring a major difference between the two viral systems.

## 4. Viral genetic determinants required for infection and pathogenesis

Although sheep are not natural hosts for BLV, studying infection and pathogenesis in this model might be informative for understanding pathogenesis pertaining to other deltaretroviruses. To circumvent the problem of genomic RNA instability, infectious proviruses were cloned and injected into sheep or calves [30,83,238,239]. Hence, infection of sheep with proviral clone 344 leads to tumor or leukemia after a mean latency period of 33 months [170]. Since the BLV provirus can be re-amplified from the tumor cells, the three conditions required to fulfill Koch's postulate are demonstrated (i.e. the cloning of the virus, the analysis of its pathogenicity, and its re-isolation from the lesions), clearly establishing viral causality in leukemia/lymphoma.

Among other isolates, clone 395 is deficient for infectivity *in vivo*, due to the presence of a E-to-K mutation at codon 303 of the Tax protein [83,184,186]. In cell culture, transfection of provirus 395 yields reduced levels of Tax activity (~ 10 % of wild-type) although the amount of major capsid protein p24 expressed inside the cells and in the supernatant remains unaffected. Adequate levels of transactivation are required for infectivity *in vivo *supporting the notion that *tax *is an essential gene.

The injection of sheep with provirus 344 fulfills all the requirements of a model system linking fields as diverse as molecular biology, virology and pathogenesis. Therefore, clone 344 has been used to construct a series of derivative proviruses harboring mutations or deletions in different parts of the genome. As expected, large deletions within the structural or enzymatic *gag*, *pol *or *env *genes destroy infectivity *in vivo *[83,112]. Interestingly, co-infection of sheep with two defective recombinants can generate a replication-competent and pathogenic virus by homologous recombination *in vivo*. As mentioned earlier (see section 2), several residues/regions in the viral genome are essential for infection: E303 of Tax, Y197 of the TM ITAM motifs and the MHR domain of CA [112,162]. Considering that genetic information is highly condensed in the proviral genome, it is surprising to identify a large domain within the provirus that is dispensable for infectivity *in vivo*. Indeed, the deletion of the region which expands from the end of the *env *gene to the splice acceptor site of the *tax/rex *mRNA does not impair infectivity ([83] and unpublished results). Since these sequences correspond respectively to the third and second exons of the R3 and G4 mRNAs, it appears that these genes are not essential for infectivity *in vivo*. Similar conclusions were drawn from HTLV mutant proviruses deleted in the ORFs encoding the p12^I ^and p13^II^/p30^II ^orthologs of R3 and G4 [240-242]. Importantly, the R3/G4 deletion greatly interferes with the efficiency of BLV propagation and restricts pathogenesis [170,172]. Very recently, however, one out of 20 sheep infected with a R3/G4 mutant developed a lymphoma after 7.5 years of latency, demonstrating that the deleted sequences are not strictly required for pathogenesis (Florins et al, in preparation). It does remain that the integrity of the R3/G4 genes significantly contributes to disease frequency and latency (see Table 1).

**Table 1:** Summary of unexpected conclusions deduced from the BLV/sheep model

Fusion-deficient viruses can propagate at wild type levels
A transformation-deficient tax mutant is leukemogenic in sheep
A large region between the env and R3 genes is dispensable for infectivity and viral spread
Deletion of R3/G4 affects but does not abrogate pathogenicity
Increasing LTR promoter activity decreases viral spread

The BLV 344/sheep system has been instrumental for unraveling determinants of the viral replication cycle. Binding of the viral envelope complex to the target cell membrane leads to a process of fusion, allowing subsequent viral entry. The fusion mechanism can be reproduced *in vitro *by co-cultivation of fibroblasts or lymphocytes expressing Env proteins at their surface and target cells like CC81, leading to polykaryocytosis [156]. The fusion process is mediated by the oblique insertion of the TM aminoterminal peptide into the lipid bilayer of the cell membrane. Forcing the peptide to adopt a parallel orientation by mutation abrogates fusion in cell culture and infectivity *in vivo *[83]. In contrast, replacement of the peptide by the corresponding residues derived from SIV (simian immunodeficiency virus) yields a fully fusion-competent envelope. However, a virus carrying this mutation lacks infectivity, suggesting that additional constraints are operative *in vivo*. What is even more surprising is that TM mutants (i.e. A60V and A64S) that are deficient for cell fusion *in vitro *nevertheless support viral infectivity *in vivo *[154]. And, very unexpectedly, these mutant viruses can propagate at wild-type levels and are pathogenic in sheep (see Table 1). Since the A60V and A64S mutants are also impaired for SU/TM interaction, it seems that integrity of the envelope complex is not strictly required *in vivo*. If the cell fusion processes *in vitro *and *in vivo *are indeed impaired equally by these mutations, then the findings offer the unexpected suggestion that BLV replicates by mitotic division of the infected cell rather than by de novo envelope-cell receptor mediated infectious cycle.

The *tax *gene is assumed to be a major factor required for viral replication and pathogenesis. Tax activates LTR-directed transcription and immortalizes primary cells in culture [190,191,199,202,243]. The two activities of Tax can be dissociated; for example, mutations in the zinc finger abrogate transactivation without altering immortalization [194]. Conversely, substitution of the two major phosphorylation sites in Tax does not alter its transcriptional activity but destroys its oncogenicity in REF cells [200]. As illustrated by the defect seen with provirus 395, Tax's transactivation activity is required for viral infectivity *in vivo*. In contrast, a provirus (Tax106+293) harboring mutated phosphorylation sites remains infectious and propagates at wild-type levels in sheep. In addition, the Tax106+293 mutant is pathogenic despite a loss in its ability to transform primary cells *in vitro *[201] (see Table 1). These findings suggest that a deficiency in Tax oncogenic potential as revealed by the REF immortalization does not correlate with leukemogenesis *in vivo*.

As previously mentioned, the BLV transcriptional promoter located in the 5' LTR contains suboptimal binding sequences for the CREB transcription factor. Remarkably, the cyclic-AMP responsive site (CRE) consensus "TGACGTCA" is never strictly conserved in any viral 21 bp element which invariably contains an imperfect substitution (for example, AGACGTCA, TGACGGCA, TGACCTCA). Restoring a perfect CRE sequence into the promoter increases LTR (long terminal repeat) promoter activity, as expected [88]. However, the proviral loads are drastically reduced in sheep infected with a virus harboring this type of change (see Table 1). It is tempting to speculate that BLV may have evolved a self-attenuating process (perhaps for purposes of escaping immunosurveillence) which encourages the virus to maintain a less active promoter through suboptimal use of the CRE-dependent pathway. If this speculation is correct, then one thought is that transcriptional repression of viral expression may be a key factor which regulates viral persistence and spread. As mentioned in section 2, the activity of the viral LTR is also thought to be regulated by E-box motifs which overlap the CRE sites [88,89]. However, an E-box mutant virus is infectious, replicates to wild-type levels and is pathogenic in sheep. These observations question the clear significance of the E-box motif *in vivo *[88] (and unpublished results).

Collectively the experimental findings from BLV research emphasize the dichotomy between subgenomic *in vitro *results and counterpart findings achieved using replicating viruses *in vivo; *they reinforce the critical need to perform pathogenesis studies *in vivo*.

## 5. Mecanisms of leukemogenesis

### BLV is an exogenous virus which integrates randomly in the cellular genome

Viral infection is followed by a polyclonal expansion of a large and diverse population of lymphocytes harboring one to five integrated proviruses [122,123,244-246]. At later stages, a few cell clones predominate and the population evolves towards monoclonality in assays of viral integration. Proviral integration thus appears to be mandatory for the viral life cycle, although each integration event may not be perfect and on occasions some sequences are deleted [183,184,247]. In fact, these relatively frequent deletions typically affect the central structural genes and yield dead-end viruses that are unable to replicate *in vivo *[83,184]. The emergence of these deletants might be a fortuitous consequence of viral replication following mistakes during reverse transcription, recombination or integration. However, the frequency at which these deletions occur in tumor samples suggests that they provide a selective advantage to the infected cell. In rare cases, it is even possible that a deleted provirus is the sole integrant within the host cell genome. However, it appears that at least one copy of the full-length BLV proviral genome is maintained in each animal throughout the course of the disease [185]. Whether these replication-competent viruses complement the deletants, as observed for spleen focus-forming virus, is unknown. The defect within the proviruses is a consequence either of large deletions or of point mutations, but is not due to insertion of cellular sequences [248,249]. Finally, BLV provirus integrates at random sites and, therefore, does not transform cells by insertional mutagenesis, as observed in ALV-induced tumors (Avian Leukosis Virus) [250,251].

### Low levels of viral expression are detected in vivo

An apparent paradox of BLV infection is that leukemogenesis proceeds in the absence of viral expression. In fact, lack of expression pertains to the large majority of detectable virus-infected cells [252,253]. The first evidence is that BLV virions or viral proteins cannot be directly detected in the peripheral blood by any currently available technique (ELISA, flow cytometry, immunoprecipitation or western blotting). Second, viral transcripts from peripheral blood lymphocytes or tumors can only be amplified by means of very sensitive RT-PCR techniques [79,180,254,255]. Third, using flow cytometry cell sorting and subsequent RT-PCR, only about one B lymphocyte out of 10,000 is found to express tax/rex mRNA during persistent lymphocytosis. Fourth, only rare cells in the peripheral blood (1 in 50,000) contain enough BLV transcripts to be readily identified by *in situ *hybridization [256,257]. A potential repression mechanism of viral expression involves a plasma factor related to fibronectin [258-260] and inhibited by a platelet lysate [261]. However, expression of BLV in samples of whole blood from BLV-infected cattle is activated immediately upon incubation at 37°C in the absence of any exogenous factors except for anticoagulants or the removal of blood cells from plasma [262].

As early as in 1969, Miller [69] showed that cultivation of infected peripheral blood mononuclear cells leads to expression of viral antigens. This process has been extensively characterized to identify the involved pathways: it appears that concanavalin A [263,264], phytohemagglutinin (PHA) [256,265], lipopolysaccharide (LPS) [179,266], phorbol esters (PMA) and calcium ionophores [24,267,268] activate viral protein synthesis. The presence of T cells increases [264,269,270], but is not strictly required for viral expression by the infected B lymphocytes. As revealed by a series of relatively specific inhibitors, the metabolic pathways involved in viral expression include protein kinase C, calmodulin and intracellular calcium mobilisation. More physiological stimuli of viral expression include cross-linking of membrane IgM or interactions with CD40 ligand, mimicking BCR and T cell activation, respectively [243,256,268]. Finally, viral transcription is activated by components of fetal bovine serum.

Altogether, these data indicate that viral expression can be augmented by molecules that mimic B cell activation by immune cells. As presented in this paragraph, the traditional and dogmatic model postulates that cells are latently infected and express viral proteins only upon transient *ex vivo *cell culture. This concept faces a series of objections and we propose another model in section 6.

### Altered gene expression of cytokines

#### Interleukins: IL2, IL6, IL10 and IL12

A first described cytokine network interconnects interleukin-10 (IL-10), viral expression and B-cell proliferation in BLV-infected cattle. IL-10 mRNA is over expressed in cows with persistent lymphocytosis [271,272]. In cell culture of PBMCs, IL-10 inhibits expression of COX-2 as well as antigen-specific cell proliferation. IL-10 suppresses synthesis of a macrophage-derived COX-2 product, prostaglandin E_2_, that stimulates virus expression [273,274].

Although reported data on IL2 expression during the course of BLV-infection are discordant, it is agreed that elevated levels of this cytokine are synthesized in mitogen-stimulated PBMCs from asymptomatic and PL cattle [225,264,275-278]. Furthermore, in isolated B lymphocytes from PL cows, IL-2 increases viral CA protein synthesis, IL-2 receptor expression, and triggers proliferation.

T cells isolated from lymph nodes and peripheral blood of BLV-infected cattle express IL-2 mRNAs [279]. However, the amounts of IL-2 mRNAs are significantly reduced in CD4+ T cells from PL cows as compared to controls; on the other hand, no significant differences in the frequencies of CD4+ T cells expressing these cytokine mRNAs are observed.

Although IL-6 mRNAs are barely detectable in fresh B cells from PL cows, transcripts encoding this cytokine are strongly and rapidly upregulated after cell culture [280]. Furthermore, levels of IL-6 are significantly higher in the sera of BLV infected cows with PL as well as in PBMC cultures following *in vitro *exposure to BLV antigens [281]. When exogenous IL-6 is added to infected cells *in vitro*, viral expression is strongly suppressed, suggesting that IL-6 plays a contributory role to viral latency.

Finally, elevated levels of IL-12 in asymptomatic and PL cattle are expressed by mitogen-stimulated PBMC [282]. However, IL-12 p40 mRNA expression is significantly decreased in PL cattle compared to aleukemic animals [283].

#### TNFα

The role of tumour necrosis factor alpha (TNFα) in BLV replication has clearly been demonstrated in TNF^-/- ^knockout mice [284]. In sheep inoculated with BLV, expression of TNFα receptor type 1 mRNA (TNF-R1) is down-regulated while TNF-R2 mRNA remains constant. BLV-infected PBMCs express membrane-bound TNFα and proliferate in response to TNFα [285,286]. Furthermore, TNFα expression is higher in sheep that resist BLV infection after vaccination [287].

In BLV-infected cattle, the mean mRNA expression level for TNF-α is higher in the spontaneously proliferating and antigen-induced PBMC population [271,272,281,288]. When exogenous TNF-α is added to BLV infected cells *in vitro*, viral expression is strongly suppressed. Cells isolated from PL cattle exhibit increased proliferative responses in the presence of recombinant bovine TNF-α [289] and express significantly higher TNF-R2 mRNA levels although no difference is found in TNF-R1 mRNA levels. Most cells expressing TNF-R2 are CD5+ or sIgM+ cells and are less prone to TNFα-induced apoptosis [285].

#### IFN-γ

As expected by its antiviral activity, recombinant bovine IFN-γ suppresses replication of ovine BLV-infected cells *in vitro *[290]. In addition, sheep, which show augmented mRNA expression of IFNγ, have lower proviral loads [291]. When BLV-infected cattle are inoculated intraperitoneally with recombinant bovine IFN-γ, γδ T cells increase soon after a period of transient fever whereas the number of BLV-infected B lymphocytes remains low during one week [292]. This experiment thus directly illustrates the potency of IFNγ to inhibit viral spread.

IFNγ mRNA is detected in the T-cell population isolated from lymph nodes of BLV-infected cattle [279,282]. In PBMCs, IFN-γ mRNA expression increases 4 weeks after infection [293] but the antiviral activity remains intriguingly unaffected [294]. In aleukemic cattle, IFNγ mRNA expression is significantly increased compared to those in cattle with persistent lymphocytosis [283]. Furthermore, IFN-γ is elevated in pokeweed mitogen-stimulated cells from asymptomatic cattle but not from PL animals [282].

Recently, another form of interferon, IFN-τ, was reported as a potential anti-viral protein in cattle [295].

### Host cell genetics

BLV-induced leukemogenesis is preceded by a long lasting chronic disease characterized by accumulation of genetic modifications such as mutation of p53 within the host genome [296-299]. Approximately half of the solid tumors induced by BLV in cattle contain a mutated p53 gene while very few mutations are found in preneoplastic B cells. These mutations interfere with essential p53 functions required for transactivation and suppression of cell growth [299]. In addition, the ratio of Bcl2 to Bax which is believed to predetermine the susceptibility to a given apoptotic stimulus is increased at advanced stages of disease in cattle [300]. In contrast, the p53 gene is not mutated at any stage of disease in sheep [301].

Tumors cells accumulate clonal abnormalities and are hyperdiploid [302]. The most common aberrations are the acquisition of additional small chromosomes, trisomy, Robertsonian translocations and isochromosome rearrangements. Whether these abnormalities are required to achieve full malignancy or are just bystanders of transformation is currently unknown. However, it is likely that these chromosomal alterations acquired during a long multistep process provide a selective growth advantage to the tumor cells.

The genetic profile of the host genome also predisposes to tumor development [303]. A major factor involved in clinical progression of BLV-infected animals is mediated by the bovine major histocompatibility complex (BoLA) [304,305]. Genetic resistance and susceptibility to persistent lymphocytosis (PL), have been mapped to structural motifs in bovine MHC DRB3 (class II) alleles [306]. Haplotype DQA*12-DQB*12-DRB2*3A-DRB3.2*8 is associated with a risk factor for subclinical progression to PL in BLV-infected animals, whereas DQA*3A-DQB*3A-DRB2*2A-DRB3.2*11 correlates with resistance [307]. Animals with the PL-resistance associated DRB3.2*11 allele have significantly fewer BLV-infected B cells than do age- and seroconversion-matched cows with DRB3 alleles associated with susceptibility to PL [308]. Furthermore, another polymorphism in the promoter region of TNF-α gene (-824G allele) may contribute to the progression of lymphoma in BLV-infection [309]

In sheep, genetic predisposition to development of leukemia correlates with a particular MHC-II DRB1 allele. The Arg-Lys (RK) and the Ser-Arg (SR) at positions 70/71 of the OLA-DRβ1 domain are associated with resistance and susceptibility, respectively, to development of lymphoma [310]. Higher levels of IFN-γ are found in animals with RK/RK genotype [311] most probably modulating disease progression.

The susceptibility to the polyclonal expansion of BLV-infected B lymphocytes is thus associated with specific alleles of the major histocompatibility complex system.

### Host humoral and cytotoxic immune responses

Natural or iatrogenic transmission of BLV primarily involves the transfer of infected cells via blood or milk [8,308]. The processes occurring after this primary infection still remain obscure. One of the earliest indications of infection is the onset of a humoral anti-viral response at about 1–8 weeks post-inoculation [308,312,313]. Antibodies recognizing epitopes from structural (envelope gp51 and capsid p24) and regulatory proteins (Tax and Rex) are synthesized at high titers. Some of these antibodies are directly lytic for BLV-producing cells [314]. The level of antibody-mediated cytolytic activity increases with progression of the disease towards the acute phase [315].

Almost concomitant with the early seroconversion period, cytotoxic T-lymphocytes (CTL) specific for Tax and Envelope epitopes appear in the peripheral blood [147,285]. Compared to humans, a peculiarity of cattle is that γδ T-lymphocytes are major players in this cytotoxic response [316]. BLV infection also triggers both a virus-dependent and a virus-independent CD4 helper T cell response [144,317,318].

It thus appears that a very active humoral and cytotoxic immune response is initiated soon after BLV infection (reviewed in [285,319]). Importantly, these anti-viral activities amplify and persist throughout the animal's life indicating that the immune system is permanently stimulated by BLV antigens. The persistence of this immune response is relatively unexpected for a chronic infection, at least if associated with a latent virus. However, cytotoxic and helper associated functions weaken in BLV-infected animals, as the disease progresses, as supported by a lower spontaneous recovery from *Trichophyton verrucosum *[285,320]

## 6. Cell dynamics of viral infection

### Is BLV silent?

Although BLV expression is detected in a minority of viable cells, a strong cytotoxic and humoral immune response is induced within the infected host. Experimental evidence (i.e. *in situ *hybridization, flow cytometry, RT-PCR) favors a model postulating that the virus is latent in the very large majority of detectable cells (i.e. those that escape from immune response and can be isolated and observed *ex vivo*). The latency of BLV *in vivo *and its reactivation upon *ex vivo *culture thus became a standing dogma. There are however a series of caveats in this model. Indeed, the maintenance of a vigorous anti-viral immune response in infected animals indicates that some degree of virus expression must occur *in vivo*. Furthermore, BLV transcription can even be detected in samples of whole blood upon incubation at 37°C without addition of any exogenous factor except anticoagulants [262]. Then, why would this process not be ongoing continuously in infected cells *in vivo*? If anticoagulants do not activate viral expression, it is unlikely, although not impossible, that the simple removal of blood would be sufficient to induce BLV transcription. Alternatively, we favor the idea that viral expression occurs permanently in a subpopulation of infected cells, which are very efficiently killed by the immune system. The cytotoxic and humoral responses are however unable to destroy cells in which viral transcription is completely silenced.

### How does the virus replicate?

Viral replication occurs via the replicative cycle after expression of virions able to infect novel target cells. Alternatively, the integrated proviruses can expand by mitosis of the host cell by a process referred to as clonal expansion [321]. Semiquantitative inverse PCR amplification of the cellular sequences flanking the BLV provirus has revealed that the viral load results almost exclusively from clonal expansion of infected cells [246]. Importantly, the premalignant cellular clones from which the tumor originates can be detected as early as a few weeks after experimental infection. In fact, the latency period preceding onset of leukemia/lymphoma is characterized by a fluctuation in the abundance of different cellular clones harboring an integrated provirus. Malignancy of a given clone correlates with the accumulation of somatic mutations revealing a decrease in the genetic stability of the expanding infected cell. During the asymptomatic phase, most of the proviral load is sustained by mitosis of the infected cell. Efficient virus replication and infection of new target cells via virions and/or virological synapses seem to occur mostly, if not almost exclusively, during a very short period following viral inoculation (so-called primary infection). However, it is still possible that the replicative cycle is ongoing continuously but the net outcome of this process does not contribute significantly to the observed viral load, because of an efficient immune response.

Two key and related questions remain to be solved: why is the abundance of the infected cell clones fluctuating? And what is the driving force of the clonal expansion process? Based on the extensively described oncogenic properties of Tax, our tenet is that this virally encoded protein triggers cell proliferation.

### Is BLV inhibiting apoptosis?

When peripheral blood mononuclear cells from BLV-infected sheep are transiently maintained in culture for a few hours, the levels of B cell apoptosis are reduced compared to normal controls [322,323]. The most straightforward interpretation is that BLV interferes with spontaneous apoptosis of B lymphocytes. This process requires at least in part a caspase 8-dependent pathway regardless of viral infection [324]. Pharmaceutical depletion of reduced glutathione (namely, gamma-glutamyl-L-cysteinyl-glycine [GSH]) by using ethacrynic acid or 1-pyrrolidinecarbodithioic acid specifically counters the inhibition of spontaneous apoptosis conferred indirectly by protective BLV-conditioned media; conversely, exogenously provided membrane-permeable GSH-monoethyl ester restores cell viability in B lymphocytes of BLV-infected sheep. Most importantly, intracellular GSH levels correlate with virus-associated protection against apoptosis but not with general inhibition of cell death induced by polyclonal activators, such as phorbol esters and ionomycin. Similar evaluations of spontaneous apoptosis in cattle yielded a very broad range of spontaneous apoptosis mainly depending on the experimental conditions [97,325,326].

**Figure 4:**
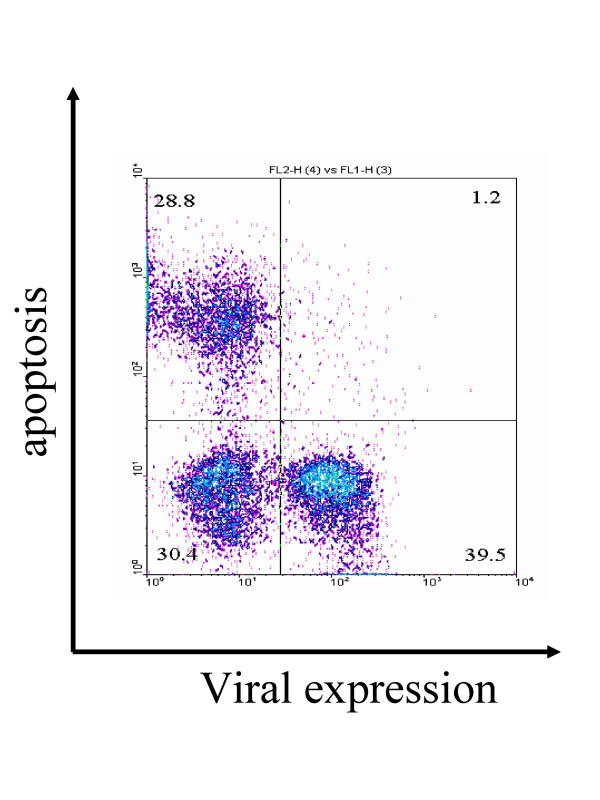
**Cells expressing CA are not prone to undergo apoptosis**. Dot plot resulting from a flow cytometry analysis of peripheral blood mononuclear cells isolated from a lymphocytic BLV-infected sheep and transiently cultivated during 24 hours. Cells were labeled with a monoclonal antibody specific for the viral major capsid protein p24gag (X axis: viral expression) and by TUNEL (Y axis: apoptosis).

A major problem with *ex vivo *experiments is that it is never possible to perfectly replicate the situation prevailing *in vivo*. Even under the culture conditions that most closely mimic the natural medium (such as culture of heparin-containing blood), interpretations of data will always face experimental objections. For instance, when lymphocytes are isolated from a BLV-infected sheep and maintained for a few hours in culture, almost all cells expressing the major viral capsid protein p24^gag ^fail to undergo apoptosis (Figure 4). This observation fits well with previous reports showing that B-lymphocytes from infected sheep are less prone to apoptosis compared to control cells [322,323]. However, an alternative interpretation is that cells that spontaneously express CA antigen are cleared *in vivo *and therefore cannot be detected *ex vivo*.

### Cell kinetics in vivo

Several markers of proliferation (PCNA, KI67 and *myc*) are overexpressed in B lymphocytes from tumors and PBMCs isolated from animals with PL, suggesting an increased replicative capacity of these cells [327-329]. However, lymphocyte homeostasis is the result of a critical balance between cell proliferation and death. Disruption of this equilibrium can lead to the onset of leukemia. Thus an increase in lymphocyte number can be potentially explained by either one or both of the above parameters.

To further gain insight into the processes mediating pathogenesis, it is necessary to determine the kinetic parameters which sustain the dynamics of the different cell populations in infected animals. The proliferation rates in BLV-infected and healthy sheep were initially determined using intravenous injection of bromodeoxyuridine (BrdU). This *in vivo *approach revealed that B-lymphocytes are proliferating significantly faster in BLV-positive asymptomatic and lymphocytic sheep than in uninfected controls (average proliferation rate of 0.020 day^-1 ^versus 0.011 day^-1^), meaning that an excess of 0.9 % cells (the difference between 2 and 1.1%) are produced by proliferation each day [330]. The difference in the proliferation rates becomes even more evident at the terminal neoplastic stage of the disease (proliferation rate increases by up to tenfold). Cells in S/G2/M then also appear in the peripheral blood (our unpublished results) similar to findings documented for acute cases of human non-viral leukemia [331]. In contrast, the death rates of the BrdU-positive cells are not significantly different between aleukemic BLV-infected and control sheep.

In the natural host, BLV-infected cattle, the cell proliferation rates in asymptomatic and control animals are not significantly different [325]. Surprisingly, the PL stage is characterized by a decreased B cell turnover resulting from a reduction of cell death as well as from an overall impairment of proliferation. Paradoxically, an excess of B lymphocytes in the peripheral blood in PL animals correlates with a reduction of cell proliferation, suggesting that a mechanism of feedback regulation controls lymphocyte homeostasis. Of note, the lymphocyte turnover is also reduced in other lymphoaccumulative diseases such as, CLL (chronic lymphocytic leukemia), a B CD5+ chronic leukemia in human (J. Defoiche, submitted). The reduced dynamic parameters measured in cattle thus contrast with the accelerated kinetics observed in experimentally infected sheep. Whether these observations relate to the differences in disease acuteness in the two host species remains a tempting but still open assumption.

Cells expressing viral proteins cannot be directly observed in the peripheral blood of the infected animals at any stage of the disease. However, viral expression can be induced upon transient short term culture. Surprisingly, very few (if any) cells spontaneously synthesizing CA antigen undergo proliferation *in vivo *[325,330]. Amongst all infected cells proliferating *in vivo *as measured by BrdU uptake, none is found to express viral protein. Since lymphocytes synthesizing p24^gag ^are spared from apoptosis *ex vivo*, p24+BrdU+ double positive cells are not lost during culturing but appear to have been eliminated *in vivo*. If we postulate that viral expression and cell activation are closely linked, as widely illustrated in the literature, the lack of p24+BrdU+ double positive cells then reveals a very efficient negative selection which occurs *in vivo*. Another non-exclusive interpretation would be that only a subpopulation of infected cells is allowed to proliferate (i.e. incorporate BrdU) provided that no viral proteins are expressed. However, this model does not fit with the progressive accumulation of provirus-positive cells, if proliferation is triggered by a viral protein. What would indeed be the selective advantage of a cell carrying a completely silent provirus?

Whatever the involved mechanisms, these kinetic studies cast light onto a very active process of immune selection directed towards proliferating infected cells that express an integrated provirus.

### Lymphocyte trafficking in lymphoid organs

**Figure 5:**
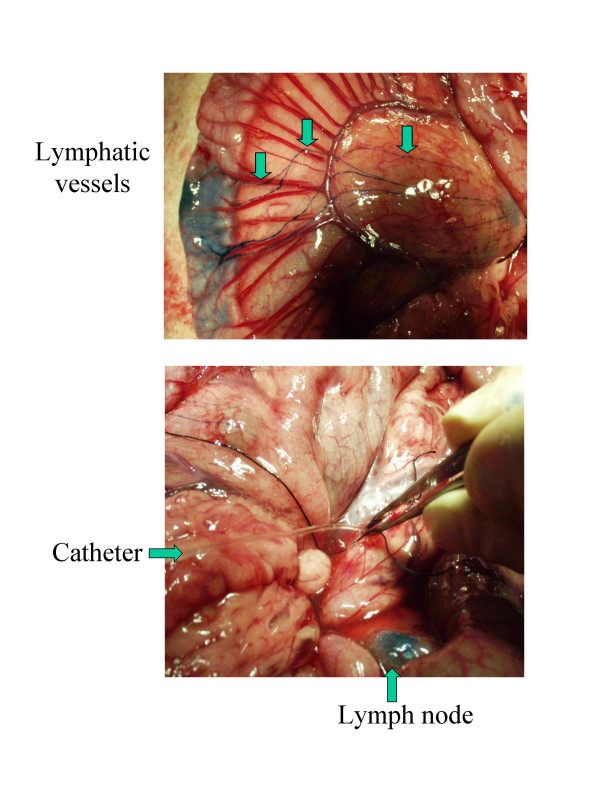
**Canulation of mesenteric lymphatic vessels**. The lymphatic vessels and the lymph nodes were colored by injection of Evans blue and dwelling catheters were inserted into the efferent lymphatics.

Homeostatic regulation of lymphocyte numbers in the peripheral blood results from a series of physiological factors, of which cell proliferation and death are only partial components. Indeed, kinetics of a cell population is also influenced by recirculation to lymphoid organs, in which proliferation is thought to primarily occur, at least under normal conditions. In this context, experiments based on BrdU kinetics lead to an apparent discrepancy: the imbalance created by the net increase in proliferation in the absence of compensating cell death is estimated at 7 % per day [330]. Since this considerable proliferation rate is not reflected by a corresponding increase in the lymphocyte numbers, other regulatory mechanisms including alteration of recirculation as well as a massive elimination of cells in other tissues could play a role. To test these hypotheses, B cell migration from blood to lymph and back from lymph to blood has been traced with the carboxyfluorescein diacetate succinimidyl ester (CFSE), a fluorescent dye that labels proteins via their NH_2 _terminal ends. Direct intravenous administration of CFSE into sheep achieves remarkable labeling indexes: more than 98% of peripheral blood leukocytes become fluorescent within seconds [332]. Since CFSE is extremely unstable in aqueous solution, labeling is very short lived, making it feasible to track lymphocyte migration from the periphery through the lymph node *in vivo*. While most studies of lymphocyte recirculation and homing have been done in rodents, the sheep model offers the opportunity to study the recirculation of lymphocytes through tissues by direct cannulation of individual lymphatic vessels [333-335] (Figure 5). Using this approach, it has been shown that B-lymphocytes from BLV-infected and control sheep recirculate with similar rates [336]. In contrast, the proportions of labeled B cells in the peripheral blood decrease significantly faster in infected sheep. Combined with another parameter (the halving of the fluorescence intensity upon cell division), it was possible to calculate proliferation and death rates [337]. These calculations indicate that B cells labeled with CFSE in the peripheral blood undergo massive destruction during chronic BLV infection of sheep [336]. Importantly, the CD11b subpopulation accounts for the difference in CFSE kinetics in BLV-infected sheep (i.e. the turnover of CD11b + B cells is increased), providing a rationale favoring the accumulation of these cells during pathogenesis.

Collectively, quantification of the dynamic parameters deduced from BrdU and CFSE kinetics shows that the excess of proliferation in lymphoid organs can be compensated by increased death of the peripheral blood cell population. An important issue that remains to be clarified is to identify the anatomic site required for this cell destruction. In this context, our recent experiments revealed that the spleen is a major lymphoid tissue regulating infected cell dynamics [338]. Indeed, the cell death rates pertaining to the peripheral blood cells of BLV-infected and control sheep are similar after splenectomy, revealing a key role of the spleen in B-lymphocyte dynamics.

**Figure 6:**
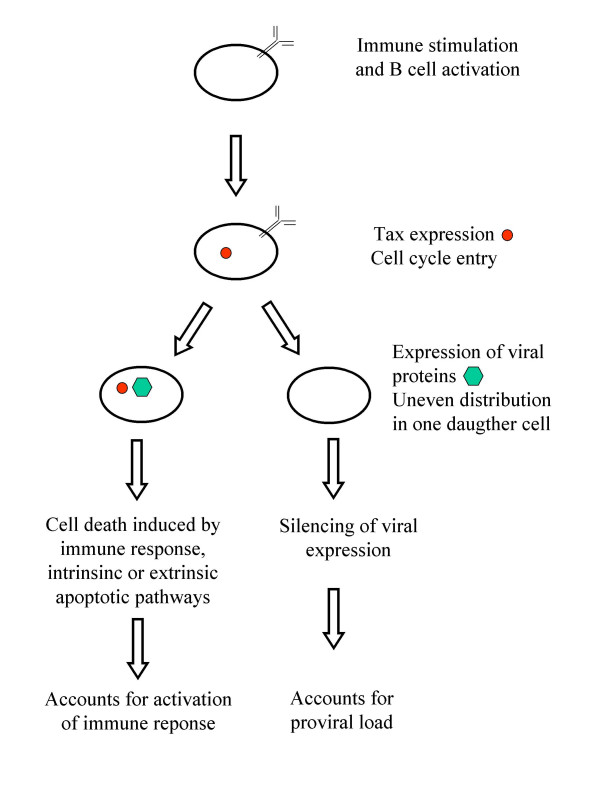
**Hypothetical mechanism of BLV replication**. Normal B cell activation and proliferation depend on a variety of immune stimuli, involving the BCR (and possibly CD5, section 3), CD40 ligand expressed by T cells, various cytokines or even autoantigens (see section 5). We hypothesize that BLV replication is initiated by these classical regulatory mechanisms because viral expression can be augmented by molecules that mimic B cell activation by immune cells. Tax expression precedes that of structural and enzymatic proteins and promotes entry into the cell cycle, providing a selective proliferative advantage of the infected cells (see section 4, The Tax transactivator). Uneven distribution of the viral proteins upon mitosis would generate two types of infected cells containing or not BLV antigens (see section 4, The envelope gene). Other processes might account for silencing of viral expression in one daugther cell such as a specific inhibition by a viral factor such as HTLV p30 or HBZ (still to be discovered for BLV). We think that BLV expression is ongoing continuously in vivo because viral transcripts are detected in whole blood immediately upon incubation at 37°C in the absence of any exogenous factors (see section 5, Low levels of viral expression are detected in vivo). Virus-positive cells would be destroyed by the immune response (see section 5, Host humoral and cytotoxic immune responses) or would undergo apoptosis via intrinsic or extrinsic pathways (section 6: Is BLV inhibiting apoptosis?). These cells would thus permanently stimulate the host's immune response. Cells in which viral expression has been shut off or lacking viral antigens after mitosis would enter a resting stage in the absence of Tax and/or immune stimulation. These cells surviving destruction by the immune response can be isolated and observed ex vivo.

Collectively, recent data show that the B lymphocyte turnover is accelerated in BLV-infected sheep. Amongst a series of plausible hypotheses that cannot be formally excluded, one of the possible models is that the increased turnover results from an activated immune response directed towards the virus. Continuous expression of viral antigens could indeed exacerbate proliferation of virus-reactive immune cells either directly or via cytokines with potential expansion of BLV-infected B lymphocytes. Excessive proliferation of uninfected B-lymphocytes in response to BLV early infection has recently been documented clearly [245]. In addition, uninfected B lymphocytes also accumulate above normal levels during persistent lymphocytosis. Whether a similar anti-viral process is also responsible for expansion of BLV-infected B cells is presently unknown. It would be interesting to determine the B cell receptor specificity of the infected B lymphocytes. For instance, IgGs of CLL leukemic B cells are targeted towards autoantigens or common bacterial infections (DNA, glycerophospholipid, lipoprotein, and polysaccharides), permitting expansion of the transformed clone. Arguments against this hypothetical mechanism of indirect viral spread include the absence of selective growth advantage conferred to the infected cells. Why would a viral antigen-specific B cell be preferentially infected by the virus? We therefore favor a model in which the virus plays an active role by continuously expressing viral proteins, like the Tax oncogene, able to promote cell proliferation and transformation (Figure 6). Tax expression could be permanent, provided that cells escape from immune response (which is a rare event), or initiated indirectly via cellular activation. Concomitantly, Tax expression would also stimulate the host's anti-viral immune response, which in turn would clear the infected cells. Escape from the immune response could be due to an uneven distribution of viral proteins between the daughter cells. Alternatively, shut off of viral expression possibly by epigenetic processes (e.g. histone acetylation, histone methylation or DNA methylation as described [100,339,340]) or involving a putative viral accessory factor (such as HBZ for HTLV [341,342]) would be a prerequisite allowing a minority of these cells to escape from immune response. Since the presence of doubly spliced tax/rex transcripts in the cytoplasm precedes that of other viral mRNAs [179], it is possible that a subpopulation of cells would exclusively express the Tax protein at least during a short interval. In this model, Tax would be the driving force providing a selective advantage and leading to clonal expansion of the infected cells. Permanent expression of Tax would not even be required in each mitotic cycle if cell activation is maintained by a hit and run mechanism as previously proposed by Mitsuaki Yoshida in 1986 [343]. A variety of other processes involving for instance NFκB may also account for ongoing mitotic replication. Finally, even if this activation cannot persist through divisions, it is easily conceivable that clonality results from a population of cells having undergone different numbers of mitotic cycles (i.e. a "ladder pattern") instead of a classical pyramid shape of the progeny population. The heterogeneity of the telomere lengths observed in clonal populations of tumor cells supports the latter hypothesis (our unpublished results).

Since very few lymphocytes expressing viral proteins can be detected directly *in vivo*, the frequency of infected cells which survive the host immune pressure is low. Also, this process would only marginally affect the very large majority of infected cells containing a silent virus (or a modestly expressed virus). The net outcome of this model would be a global stability of the proviral loads with some fluctuations of individual clones, as revealed by long term follow-up of proviral integration sites by ligation-mediated PCR. Although the mechanism is still unknown, variations in the abundance of provirus-positive cell clones could be due to differential antigen stimulation or to modulation of the proportions of individual progeny cells to express virus. Hence, cells isolated from sheep at terminal stages of the disease lose their ability to efficiently express virus *ex vivo *even in the presence of potent polyclonal activators such as phorbol esters (our unpublished results). Ultimately, a fully transformed cell clone containing a deleted replication defective provirus can even outgrow and induce leukemia [83].

A hypothetical model, which may also apply to HTLV [344], is needed to reconcile the following experimental findings which include the oncogenic potential of Tax, the continuous stimulation of the immune system, the low levels of detectable cells expressing viral proteins *in vivo*, the apparent stability of individual proviral clones, and the dynamic parameters of cell proliferation and cell death. During BLV chronic infection, the host-pathogen interplay is characterized by a very dynamic kinetics generating equilibrium between a virus attempting to proliferate under a tight control exerted by the immune response. In this model, the virus permanently transits between a latent and transcriptionally active phase resulting in the progressive accumulation of viable infected cells. Occurrence of somatic mutations associated with genetic instability in these cells ultimately permits the outgrowth of a transformed clone, leading to leukemia.

## 7. Modulation of viral expression as therapy

In the absence of *ex vivo *cell culture, the infected lymphocytes apparently do not express any viral protein in the peripheral blood and rest in the G0/G1 phase of the cell cycle. These apparently quiescent cells may undergo spontaneous cell proliferation and express virions upon transient short-term culturing. Agents known to activate immune cells polyclonally cause an increase in the number of cells containing BLV RNA [345]. Viral expression may thus be induced by activation of the host cell after immune-mediated stimulation.

**Figure 7:**
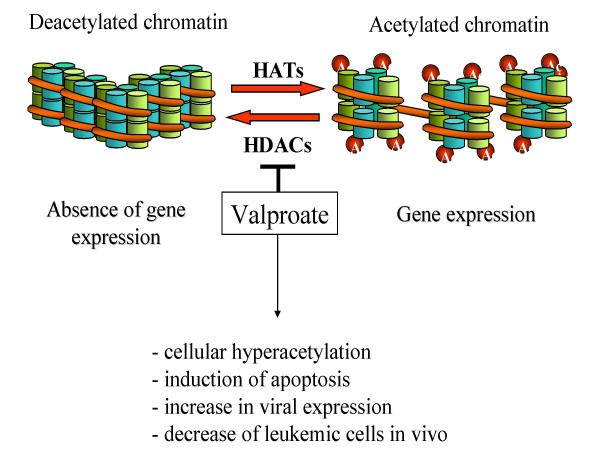
**Valproate, an inhibitor of deacetylases, activates gene transcription and induces apoptosis**. Reciprocal reactions, acetylation and deacetylation are catalyzed by histone acetyltransferase (HAT) and histone deacetylase (HDAC). Acetylation of chromatin leads to deconsendation and activates transcription of a subset of genes. Valproate induces hyperacetylation, activates BLV expression and triggers apoptosis in cell culture. Valproate treatment of sheep with leukemia/lymphoma reduces the number of tumor cells.

It is possible that an inhibitory mechanism (or the absence of a driving force) hampers viral gene expression *in vivo*. Persistence of infected cells would thus be permitted under the restrictive condition where viral proteins are not expressed (or at least at a level undetectable by the immune system). Evidence for a very strong immune response is supported by the presence of virus specific cytotoxic T cells and by high titers of cytolytic antibodies. However, the lack of viral expression in a large proportion of infected cells does not allow efficient clearance by the immune system. Concomitantly, virus infection might also correlate with inhibition of the apoptotic processes, generating a reservoir of apparently latent cells. In this context, we aimed at evaluating the therapeutic effectiveness of a strategy based on the induction of viral and cellular gene expression. Among a number of methodological approaches, modulation of chromatin condensation, which is an essential component of the gene expression process, can be achieved by interference with the level of histone acetylation [346]. This mechanism results from an intrinsic balance between the activity of two families of antagonistic enzymes, histone deacetylases (HDAC) and histone acetyltransferases, respectively removing or incorporating acetyl groups into core histones (Figure 7). Although this model is probably oversimplified, acetyl removal by HDACs restores a positive charge to the lysine residues in the histone N-terminal tails and is thought to increase the affinity of histones for DNA, leading to transcriptional repression. Conversely, impairment of HDAC function with specific HDAC inhibitors activates both cellular and viral gene transcription.

Among a growing list of HDAC inhibitors, valproate (the sodium salt of 2-propylpentanoic acid) offers a series of advantages [347]. Widely used for several decades in treating epilepsy, this short-chain fatty acid has a very high bioavailability, exhibits very low toxicity in adults and, with a half life of 16–17 hours, has suitable pharmacokinetic properties *in vivo*. Therefore, valproate has been selected to evaluate the effectiveness of gene activation chemotherapy in leukemic sheep. Indeed, valproate effectively activates BLV gene expression in transient transfection experiments and in short-term cultures of primary B-lymphocytes [339]. *In vivo*, valproate administration, in the absence of any other cytotoxic drug, is efficient for the treatment of leukemia/lymphoma in sheep, demonstrating the proof-of-concept of a therapy that targets the expression of viral and/or cellular genes. Interestingly, over a long term period, valproate treatment alters neither the cell numbers in control sheep nor other lymphocyte populations in BLV-infected animals, revealing a relative innocuousness of the therapy.

The mechanism by which valproate specifically decreases the number of leukemic cells remains to be determined. Amongst numerous hypotheses, we propose a model based on transient activation of cellular and/or viral expression leading to apoptosis by intrinsic (for instance dependent on mitochondrial regulated checkpoints) or extrinsic mechanisms related to membrane-bound receptors (Fas, TRAIL,) [348-350]. It is possible that the involved mechanisms are completely independent of viral infection as indicated by our ongoing experiments in mesothelioma and chronic lymphocytic leukemia cells (F. Vandermeers and A. Bouzar). Alternatively, a very attractive model would include the induction of viral expression and destruction by the immune response.

Based on the numerous similarities between BLV and HTLV, we propose to investigate the therapeutic potential of a valproate treatment in TSP/HAM and ATL. Indeed, there are presently no satisfactory therapies for these diseases so far. Palliative treatments for HAM/TSP include steroids to decrease inflammation [351-353]. Attempts to treat HAM/TSP by interfering with cell invasion into the CNS using inhibitors of matrix metalloproteinases [354] have been unsuccessful. Similarly, other strategies aimed at inhibiting cell activation and/or viral replication with cytokines (IFN-α or -β) [353] or anti-viral compounds [355-358] did not show sufficient efficacy. ATL patients have a very poor prognosis because of intrinsic chemoresistance and severe immunosuppression. Non-targeted combinations of chemotherapy (CHOP) yield primary responses but lack a significant effect on complete remission and survival [359]. Besides promising new drugs such as arsenic trioxide and proteasome inhibitors, additional therapies are needed. In this context, valproate-based gene activation therapy should be considered.

## 8. Conclusion

BLV is the etiologic agent of enzootic bovine leukemia in cattle, a disease which (depending on the part of the world) is either eradicated or simply ignored. However, the experimental infection of sheep with BLV provides an interesting model of leukemogenesis. In particular, we review here the viral genetic determinants required for pathogenesis and present recent understanding of the homeostatic regulation of leukemic B lymphocytes in BLV-infected sheep. We also propose a novel therapeutic concept targeting the expression of viral genes and involving the immune response. This strategy might also hold promise for treating adult T cell leukemia (ATL) or human T-lymphotropic virus-associated myelopathy/tropical spastic paraparesis (HAM/TSP), diseases for which no satisfactory treatment currently exists.

## List of abbreviations

ALV: avian leukosis virus

AP3: adaptator-related protein complex 3

ATF: activating transcriptor factor

BCR: B-cell receptor

BLV: bovine leukemia virus

BrdU: bromodeoxyuridine

CA: capsid protein

CaMKIV: calmodulin-depend kinase IV

CDR: complementary determining region

CFSE: carboxyfluorescein diacetate succinimidyl ester

CLL: chronic lymphocytic leukemia

CRE: cyclic-AMP responsive element

CREB: CRE binding protein

CTL: cytotoxic T lymphocyte

EBL: enzootic bovine leukemia

FPPS: farnesyl pyrophosphate synthetase

HDAC: histone deacetylase

HIV: human immunodeficiency virus

HTLV-1: human T-lymphotropic virus type 1

IFN: interferon

IL: interleukin

IN: integrase

IRF: interferon regulatory factor

ITAM: immunoreceptor tyrosine-based activation motif

ITIM: immunoreceptor tyrosine-based inhibition motif

LTR: long terminal repeat

NC: nucleocapsid protein

NF-κB: nuclear factor κB

MA: matrix protein

MHC: major histocompatibility complex

PBMC: peripheral blood mononuclear cell

PCNA: proliferating cell nuclear antigen

PKA: protein kinase A

PL: persistant lymphocytosis

PTLV: primate T-lymphotropic virus

RBD: receptor binding domain

REF: rat embryo fibroblast

RT: reverse transcriptase

SDF-1: stromal cell-derived factor-1

SNV: spleen necrosis virus

SU: surface protein

TM: transmembrane protein

TNFα:tumor necrosis factor α

TTP: tristetraprolin

TxRE: tax responsive element

USF: upstream stimulating factor

## Competing interests

The author(s) declare that they have no competiting interests.

## Authors' contributions

NG, FV, HB, AN and AB carry out experiments of gene activation therapy on BLV-infected animals. AF, MB, CB and JD worked on cell dynamics and viral infection. NG and LW drafted the manuscript and AB, MR and RK participated to its design and helped to draft it.

All authors read and approved the final manuscript.

## References

[B1] Leisering A (1871). Ber Vet -Wes Kgr Sachen.

[B2] Burny A, Cleuter Y, Kettmann R, Mammerickx M, Marbaix G, Portetelle D, Van den BA, Willems L, Thomas R (1987). Bovine leukaemia: facts and hypotheses derived from the study of an infectious cancer. Cancer Surv.

[B3] Olson C, Miller J (1987). Enzootic bovine leukosis and bovine leukemia virus Martinus Nijhoff Publishing, Boston.

[B4] Willems L, Burny A, Dangoisse O, Collete D, Dequiedt F, Gatot JS, Kerkhofs P, Lefebvre L, Merezak C, Portetelle D, Twizere JC, Kettmann R (1999). Bovine leukemia virus as a model for human T-cell leukemia virus. Current Topics in Virology.

[B5] Meas S, Usui T, Ohashi K, Sugimoto C, Onuma M (2002). Vertical transmission of bovine leukemia virus and bovine immunodeficiency virus in dairy cattle herds. Vet Microbiol.

[B6] Ferrer JF, Piper CE (1978). An evaluation of the role of milk in the natural transmission of BLV. Ann Rech Vet.

[B7] Hopkins SG, DiGiacomo RF (1997). Natural transmission of bovine leukemia virus in dairy and beef cattle. Vet Clin North Am Food Anim Pract.

[B8] Mammerickx M, Portetelle D, de Clercq K, Burny A (1987). Experimental transmission of enzootic bovine leukosis to cattle, sheep and goats: infectious doses of blood and incubation period of the disease. Leuk Res.

[B9] Kohara J, Konnai S, Onuma M (2006). Experimental transmission of Bovine leukemia virus in cattle via rectal palpation. Jpn J Vet Res.

[B10] Brenner J, Rosenthal I, Bernstein S, Trainin Z (1990). The fat content of milk from dairy cattle infected with bovine leukosis virus. Vet Res Commun.

[B11] Da Y, Shanks RD, Stewart JA, Lewin HA (1993). Milk and fat yields decline in bovine leukemia virus-infected Holstein cattle with persistent lymphocytosis. Proc Natl Acad Sci U S A.

[B12] Huber NL, DiGiacomo RF, Evermann JF, Studer E (1981). Bovine leukemia virus infection in a large Holstein herd: prospective comparison of production and reproductive performance in antibody-negative and antibody-positive cows. Am J Vet Res.

[B13] Jacobs RM, Heeney JL, Godkin MA, Leslie KE, Taylor JA, Davies C, Valli VE (1991). Production and related variables in bovine leukaemia virus-infected cows. Vet Res Commun.

[B14] Pollari FL, Wangsuphachart VL, DiGiacomo RF, Evermann JF (1992). Effects of bovine leukemia virus infection on production and reproduction in dairy cattle. Can J Vet Res.

[B15] Wu MC, Shanks RD, Lewin HA (1989). Milk and fat production in dairy cattle influenced by advanced subclinical bovine leukemia virus infection. Proc Natl Acad Sci U S A.

[B16] Kurdi A, Blankenstein P, Marquardt O, Ebner D (1999). Study on the presence of BLV infection in a dairy herd in Syria by using serological and virological tests. Berliner und Munchener Tierarztliche Wochenschrift.

[B17] Motton DD, Buehring GC (2003). Bovine leukemia virus alters growth properties and casein synthesis in mammary epithelial cells. J Dairy Sci.

[B18] Trono KG, Perez-Filgueira DM, Duffy S, Borca MV, Carrillo C (2001). Seroprevalence of bovine leukemia virus in dairy cattle in Argentina: comparison of sensitivity and specificity of different detection methods. Vet Microbiol.

[B19] Ott SL, Johnson R, Wells SJ (2003). Association between bovine-leukosis virus seroprevalence and herd-level productivity on US dairy farms. Prev Vet Med.

[B20] Rhodes JK, Pelzer KD, Johnson YJ (2003). Economic implications of bovine leukemia virus infection in mid-Atlantic dairy herds. J Am Vet Med Assoc.

[B21] Bendixen HJ (1967). Epizootiology, diagnosis and control of bovine leukosis. Bull Off Int Epizoot.

[B22] Altanerova V, Holicova D, Kucerova L, Altaner C, Lairmore MD, Boris-Lawrie K (2004). Long-term infection with retroviral structural gene vector provides protection against bovine leukemia virus disease in rabbits. Virology.

[B23] Bondzio A, Abraham-Podgornik A, Blankenstein P, Risse S (2001). Involvement of intracellular Ca2+ in the regulation of bovine leukemia virus expression. Biol Chem.

[B24] Camargos MF, Pereda A, Stancek D, Rocha MA, Reis JK, Greiser-Wilke I, Leite RC (2006). Molecular characterization of the env gene from Brazilian field isolates of Bovine Leukemia Virus. Virus Genes.

[B25] Ferens WA, Cobbold R, Hovde CJ (2006). Intestinal Shiga toxin-producing Escherichia coli bacteria mitigate bovine leukemia virus infection in experimentally infected sheep. Infect Immun.

[B26] Kerkhofs P, Gatot JS, Knapen K, Mammerickx M, Burny A, Portetelle D, Willems L, Kettmann R (2000). Long-term protection against bovine leukaemia virus replication in cattle and sheep. J Gen Virol.

[B27] Murovska MF, Chernobayeva LG, Miroshnichenko OI, Tomsons VP, Konicheva VV, Ivanova SV, Tikhonenko TI (1992). An investigation of the effect of antisense RNA gene on bovine leukaemia virus reproduction in cell culture. Vet Microbiol.

[B28] Kucerova L, Altanerova V, Altaner C, Boris-Lawrie K (1999). Bovine leukemia virus structural gene vectors are immunogenic and lack pathogenicity in a rabbit model. J Virol.

[B29] Okada K, Nakae N, Kuramochi K, Yin SA, Ikeda M, Takami S, Hirata T, Goryo M, Numakunai S, Takeshima SN, Takahashi M, Tajima S, Konnai S, Onuma M, Aida Y (2005). Bovine leukemia virus high tax molecular clone experimentally induces leukemia/lymphoma in sheep. J Vet Med Sci.

[B30] Watarai S, Aida Y, Tajima S, Kakidani H, Onuma M, Kodama H, Tana (2001). Growth inhibition of cancer cells by co-transfection of diphtheria toxin A-chain gene plasmid with bovine leukemia virus-tax expression vector. Microbiol Immunol.

[B31] Usui T, Konnai S, Tajima S, Watarai S, Aida Y, Ohashi K, Onuma M (2003). Protective effects of vaccination with bovine leukemia virus (BLV) Tax DNA against BLV infection in sheep. J Vet Med Sci.

[B32] Reichert M, Cantor GH, Willems L, Kettmann R (2000). Protective effects of a live attenuated bovine leukaemia virus vaccine with deletion in the R3 and G4 genes. J Gen Virol.

[B33] Rola M, Kuzmak J (2002). The detection of bovine leukemia virus proviral DNA by PCR-ELISA. J Virol Methods.

[B34] Marin C, de Lopez NM, Alvarez L, Lozano O, Espana W, Castanos H, Leon A (1978). Epidemiology of bovine leukemia in Venezuela. Ann Rech Vet.

[B35] Meas S, Seto J, Sugimoto C, Bakhsh M, Riaz M, Sato T, Naeem K, Ohashi K, Onuma M (2000). Infection of bovine immunodeficiency virus and bovine leukemia virus in water buffalo and cattle populations in Pakistan. J Vet Med Sci.

[B36] Singh CM, Singh B, Parihar NS (1973). Pulmonary involvement in lymphosarcoma of Indian buffaloes. Bibl Haematol.

[B37] Altanerova V, Ban J, Altaner C (1989). Induction of immune deficiency syndrome in rabbits by bovine leukaemia virus. AIDS.

[B38] Onuma M, Wada M, Yasutomi Y, Yamamoto M, Okada HM, Kawakami Y (1990). Suppression of immunological responses in rabbits experimentally infected with bovine leukemia virus. Vet Microbiol.

[B39] Wyatt CR, Wingett D, White JS, Buck CD, Knowles D, Reeves R, Magnuson NS (1989). Persistent infection of rabbits with bovine leukemia virus associated with development of immune dysfunction. J Virol.

[B40] Altanerova V, Portetelle D, Kettmann R, Altaner C (1989). Infection of rats with bovine leukaemia virus: establishment of a virus-producing rat cell line. J Gen Virol.

[B41] Boris-Lawrie K, Altanerova V, Altaner C, Kucerova L, Temin HM (1997). In vivo study of genetically simplified bovine leukemia virus derivatives that lack tax and rex. J Virol.

[B42] Altanerova V, Ban J, Kettmann R, Altaner C (1990). Induction of leukemia in chicken by bovine leukemia virus due to insertional mutagenesis. Arch Geschwulstforsch.

[B43] Mammerickx M, Portetelle D, Burny A (1981). Experimental cross-transmissions of bovine leukemia virus (BLV) between several animal species. Zentralbl Veterinarmed B.

[B44] Olson C, Kettmann R, Burny A, Kaja R (1981). Goat lymphosarcoma from bovine leukemia virus. J Natl Cancer Inst.

[B45] Djilali S, Parodi AL, Levy D, Cockerell GL (1987). Development of leukemia and lymphosarcoma induced by bovine leukemia virus in sheep: a hematopathological study. Leukemia.

[B46] Djilali S, Parodi AL (1989). The BLV-induced leukemia--lymphosarcoma complex in sheep. Vet Immunol Immunopathol.

[B47] Mammerickx M, Palm R, Portetelle D, Burny A (1988). Experimental transmission of enzootic bovine leukosis to sheep: latency period of the tumoral disease. Leukemia.

[B48] Zhao TM, Hague B, Caudell DL, Simpson RM, Kindt TJ (2005). Quantification of HTLV-I proviral load in experimentally infected rabbits. Retrovirology.

[B49] Derse D, Martarano L (1990). Construction of a recombinant bovine leukemia virus vector for analysis of virus infectivity. J Virol.

[B50] Graves DC, Ferrer JF (1976). In vitro transmission and propagation of the bovine leukemia virus in monolayer cell cultures. Cancer Res.

[B51] Inabe K, Ikuta K, Aida Y (1998). Transmission and propagation in cell culture of virus produced by cells transfected with an infectious molecular clone of bovine leukemia virus. Virology.

[B52] Milan D, Nicolas JF (1991). Activator-dependent and activator-independent defective recombinant retroviruses from bovine leukemia virus. J Virol.

[B53] DiGiacomo RF, Hopkins SG (1997). Food animal and poultry retroviruses and human health. Vet Clin North Am Food Anim Pract.

[B54] Perzova RN, Loughran TP, Dube S, Ferrer J, Esteban E, Poiesz BJ (2000). Lack of BLV and PTLV DNA sequences in the majority of patients with large granular lymphocyte leukaemia. Br J Haematol.

[B55] Burmeister T, Schwartz S, Hummel M, Hoelzer D, Thiel E (2007). No genetic evidence for involvement of Deltaretroviruses in adult patients with precursor and mature T-cell neoplasms. Retrovirology.

[B56] Buehring GC, Philpott SM, Choi KY (2003). Humans have antibodies reactive with Bovine leukemia virus. AIDS Res Hum Retroviruses.

[B57] Calattini S, Chevalier SA, Duprez R, Afonso P, Froment A, Gessain A, Mahieux R (2006). Human T-cell lymphotropic virus type 3: complete nucleotide sequence and characterization of the human tax3 protein. J Virol.

[B58] Mahieux R, Gessain A (2005). [New human retroviruses: HTLV-3 and HTLV-4]. Med Trop (Mars ).

[B59] Wolfe ND, Heneine W, Carr JK, Garcia AD, Shanmugam V, Tamoufe U, Torimiro JN, Prosser AT, Lebreton M, Mpoudi-Ngole E, McCutchan FE, Birx DL, Folks TM, Burke DS, Switzer WM (2005). Emergence of unique primate T-lymphotropic viruses among central African bushmeat hunters. Proc Natl Acad Sci U S A.

[B60] Rice NR, Stephens RM, Couez D, Deschamps J, Kettmann R, Burny A, Gilden RV (1984). The nucleotide sequence of the env gene and post-env region of bovine leukemia virus. Virology.

[B61] Sagata N, Yasunaga T, Ohishi K, Tsuzuku-Kawamura J, Onuma M, Ikawa Y (1984). Comparison of the entire genomes of bovine leukemia virus and human T-cell leukemia virus and characterization of their unidentified open reading frames. EMBO J.

[B62] Sagata N, Yasunaga T, Ogawa Y, Tsuzuku-Kawamura J, Ikawa Y (1984). Bovine leukemia virus: unique structural features of its long terminal repeats and its evolutionary relationship to human T-cell leukemia virus. Proc Natl Acad Sci U S A.

[B63] Dube S, Bachman S, Spicer T, Love J, Choi D, Esteban E, Ferrer JF, Poiesz BJ (1997). Degenerate and specific PCR assays for the detection of bovine leukaemia virus and primate T cell leukaemia/lymphoma virus pol DNA and RNA: phylogenetic comparisons of amplified sequences from cattle and primates from around the world. J Gen Virol.

[B64] Mamoun RZ, Morisson M, Rebeyrotte N, Busetta B, Couez D, Kettmann R, Hospital M, Guillemain B (1990). Sequence variability of bovine leukemia virus env gene and its relevance to the structure and antigenicity of the glycoproteins. J Virol.

[B65] Licursi M, Inoshima Y, Wu D, Yokoyama T, Gonzalez ET, Sentsui H (2003). Provirus variants of bovine leukemia virus in naturally infected cattle from Argentina and Japan. Vet Microbiol.

[B66] Monti G, Schrijver R, Beier D (2005). Genetic diversity and spread of Bovine leukaemia virus isolates in Argentine dairy cattle. Arch Virol.

[B67] Ferrer JF, Stock ND, Lin P (1971). Detection of replicating C-type viruses in continuous cell cultures established from cows with leukemia: effect of the culture medium. J Natl Cancer Inst.

[B68] Miller JM, Miller LD, Olson C, Gillette KG (1969). Virus-like particles in phytohemagglutinin-stimulated lymphocyte cultures with reference to bovine lymphosarcoma. J Natl Cancer Inst.

[B69] Ghysdael J, Kettmann R, Burny A (1979). Translation of bovine leukemia virus virion RNAs in heterologous protein-synthesizing systems. J Virol.

[B70] Katoh I, Kyushiki H, Sakamoto Y, Ikawa Y, Yoshinaka Y (1991). Bovine leukemia virus matrix-associated protein MA(p15): further processing and formation of a specific complex with the dimer of the 5'-terminal genomic RNA fragment. J Virol.

[B71] Katoh I, Yasunaga T, Yoshinaka Y (1993). Bovine leukemia virus RNA sequences involved in dimerization and specific gag protein binding: close relation to the packaging sites of avian, murine, and human retroviruses. J Virol.

[B72] Mansky LM, Krueger AE, Temin HM (1995). The bovine leukemia virus encapsidation signal is discontinuous and extends into the 5' end of the gag gene. J Virol.

[B73] Kurg A, Sommer G, Metspalu A (1995). An RNA stem-loop structure involved in the packaging of bovine leukemia virus genomic RNA in vivo. Virology.

[B74] Mansky LM, Wisniewski RM (1998). The bovine leukemia virus encapsidation signal is composed of RNA secondary structures. J Virol.

[B75] Mansky LM, Gajary LC (2002). The primary nucleotide sequence of the bovine leukemia virus RNA packaging signal can influence efficient RNA packaging and virus replication. Virology.

[B76] Jewell NA, Mansky LM (2005). Packaging of heterologous RNAs by a minimal bovine leukemia virus RNA packaging signal into virus particles. Arch Virol.

[B77] Wang H, Norris KM, Mansky LM (2003). Involvement of the matrix and nucleocapsid domains of the bovine leukemia virus Gag polyprotein precursor in viral RNA packaging. J Virol.

[B78] Alexandersen S, Carpenter S, Christensen J, Storgaard T, Viuff B, Wannemuehler Y, Belousov J, Roth JA (1993). Identification of alternatively spliced mRNAs encoding potential new regulatory proteins in cattle infected with bovine leukemia virus. J Virol.

[B79] Mamoun RZ, Astier-Gin T, Kettmann R, Deschamps J, Rebeyrotte N, Guillemain BJ (1985). The pX region of the bovine leukemia virus is transcribed as a 2.1-kilobase mRNA. J Virol.

[B80] Derse D, Casey JW (1986). Two elements in the bovine leukemia virus long terminal repeat that regulate gene expression. Science.

[B81] Katoh I, Yoshinaka Y, Ikawa Y (1989). Bovine leukemia virus trans-activator p38tax activates heterologous promoters with a common sequence known as a cAMP-responsive element or the binding site of a cellular transcription factor ATF. EMBO J.

[B82] Willems L, Kettmann R, Dequiedt F, Portetelle D, Voneche V, Cornil I, Kerkhofs P, Burny A, Mammerickx M (1993). In vivo infection of sheep by bovine leukemia virus mutants. J Virol.

[B83] Adam E, Kerkhofs P, Mammerickx M, Kettmann R, Burny A, Droogmans L, Willems L (1994). Involvement of the cyclic AMP-responsive element binding protein in bovine leukemia virus expression in vivo. J Virol.

[B84] Adam E, Kerkhofs P, Mammerickx M, Burny A, Kettman R, Willems L (1996). The CREB, ATF-1, and ATF-2 transcription factors from bovine leukemia virus-infected B lymphocytes activate viral expression. J Virol.

[B85] Boros IM, Tie F, Giam CZ (1995). Interaction of bovine leukemia virus transactivator Tax with bZip proteins. Virology.

[B86] Yin MJ, Gaynor RB (1996). Complex formation between CREB and Tax enhances the binding affinity of CREB for the human T-cell leukemia virus type 1 21-base-pair repeats. Mol Cell Biol.

[B87] Merezak C, Pierreux C, Adam E, Lemaigre F, Rousseau GG, Calomme C, Van Lint C, Christophe D, Kerkhofs P, Burny A, Kettmann R, Willems L (2001). Suboptimal enhancer sequences are required for efficient bovine leukemia virus propagation in vivo: implications for viral latency. J Virol.

[B88] Calomme C, Dekoninck A, Nizet S, Adam E, Nguyen TL, Van den BA, Willems L, Kettmann R, Burny A, Van Lint C (2004). Overlapping CRE and E box motifs in the enhancer sequences of the bovine leukemia virus 5' long terminal repeat are critical for basal and acetylation-dependent transcriptional activity of the viral promoter: implications for viral latency. J Virol.

[B89] Brooks PA, Nyborg JK, Cockerell GL (1995). Identification of an NF-kappa B binding site in the bovine leukemia virus promoter. J Virol.

[B90] Brooks PA, Cockerell GL, Nyborg JK (1998). Activation of BLV transcription by NF-kappa B and Tax. Virology.

[B91] Niermann GL, Buehring GC (1997). Hormone regulation of bovine leukemia virus via the long terminal repeat. Virology.

[B92] Xiao J, Buehring GC (1998). In vivo protein binding and functional analysis of cis-acting elements in the U3 region of the bovine leukemia virus long terminal repeat. J Virol.

[B93] Dekoninck A, Calomme C, Nizet S, de Launoit Y, Burny A, Ghysdael J, Van Lint C (2003). Identification and characterization of a PU.1/Spi-B binding site in the bovine leukemia virus long terminal repeat. Oncogene.

[B94] Calomme C, Nguyen TL, de Launoit Y, Kiermer V, Droogmans L, Burny A, Van Lint C (2002). Upstream stimulatory factors binding to an E box motif in the R region of the bovine leukemia virus long terminal repeat stimulates viral gene expression. J Biol Chem.

[B95] Kiermer V, Van Lint C, Briclet D, Vanhulle C, Kettmann R, Verdin E, Burny A, Droogmans L (1998). An interferon regulatory factor binding site in the U5 region of the bovine leukemia virus long terminal repeat stimulates Tax-independent gene expression. J Virol.

[B96] Merezak C, Reichert M, Van Lint C, Kerkhofs P, Portetelle D, Willems L, Kettmann R (2002). Inhibition of histone deacetylases induces bovine leukemia virus expression in vitro and in vivo. J Virol.

[B97] Nguyen TL, Calomme C, Wijmeersch G, Nizet S, Veithen E, Portetelle D, de Launoit Y, Burny A, Van Lint C (2004). Deacetylase inhibitors and the viral transactivator TaxBLV synergistically activate bovine leukemia virus gene expression via a cAMP-responsive element- and cAMP-responsive element-binding protein-dependent mechanism. J Biol Chem.

[B98] Tajima S, Tsukamoto M, Aida Y (2003). Latency of viral expression in vivo is not related to CpG methylation in the U3 region and part of the R region of the long terminal repeat of bovine leukemia virus. J Virol.

[B99] Taniguchi Y, Nosaka K, Yasunaga J, Maeda M, Mueller N, Okayama A, Matsuoka M (2005). Silencing of human T-cell leukemia virus type I gene transcription by epigenetic mechanisms. Retrovirology.

[B100] Derse D (1988). trans-acting regulation of bovine leukemia virus mRNA processing. J Virol.

[B101] Deshayes L, Levy D, Parodi AL, Levy JP (1977). Proteins of bovine leukemia virus. I. Characterization and reactions with natural antibodies. J Virol.

[B102] Gupta P, Ferrer JF (1980). Detection of a precursor-like protein of bovine leukaemia virus structural polypeptides in purified virions. J Gen Virol.

[B103] Mamoun RZ, Astier T, Guillemain B, Duplan JF (1983). Bovine lymphosarcoma: expression of BLV-related proteins in cultured cells. J Gen Virol.

[B104] Oroszlan S, Copeland TD, Henderson LE, Stephenson JR, Gilden RV (1979). Amino-terminal sequence of bovine leukemia virus major internal protein: homology with mammalian type C virus p30 structural proteins. Proc Natl Acad Sci U S A.

[B105] Uckert W, Grofova M, Beaudreau G (1984). Translational order of bovine leukemia virus gag and env gene-coded proteins. Virology.

[B106] Matthews S, Mikhailov M, Burny A, Roy P (1996). The solution structure of the bovine leukaemia virus matrix protein and similarity with lentiviral matrix proteins. EMBO J.

[B107] Copeland TD, Morgan MA, Oroszlan S (1983). Complete amino acid sequence of the nucleic acid-binding protein of bovine leukemia virus. FEBS Lett.

[B108] Morcock DR, Katakam S, Kane BP, Casas-Finet JR (2002). Fluorescence and nucleic acid binding properties of bovine leukemia virus nucleocapsid protein. Biophys Chem.

[B109] Gilden RV, Long CW, Hanson M, Toni R, Charman HP, Oroszlan S, Miller JM, Van der Maaten MJ (1975). Characteristics of the major internal protein and RNA-dependent DNA polymerase of bovine leukaemia virus. J Gen Virol.

[B110] Mager A, Masengo R, Mammerickx M, Letesson JJ (1994). T cell proliferative response to bovine leukaemia virus (BLV): identification of T cell epitopes on the major core protein (p24) in BLV-infected cattle with normal haematological values. J Gen Virol.

[B111] Willems L, Kerkhofs P, Attenelle L, Burny A, Portetelle D, Kettmann R (1997). The major homology region of bovine leukaemia virus p24gag is required for virus infectivity in vivo. J Gen Virol.

[B112] Onuma M, Tsukiyama K, Ohya K, Morishima Y, Ohno R (1987). Detection of cross-reactive antibody to BLV p24 in sera of human patients infected with HTLV. Microbiol Immunol.

[B113] Zandomeni RO, Carrera-Zandomeni M, Esteban E, Ferrer JF (1991). The trans-activating C-type retroviruses share a distinct epitope(s) that induces antibodies in certain infected hosts. J Gen Virol.

[B114] Morgan MA, Copeland TD, Oroszlan S (1983). Structural and antigenic analysis of the nucleic acid-binding proteins of bovine and feline leukemia viruses. J Virol.

[B115] Yoshinaka Y, Katoh I, Copeland TD, Smythers GW, Oroszlan S (1986). Bovine leukemia virus protease: purification, chemical analysis, and in vitro processing of gag precursor polyproteins. J Virol.

[B116] Llames L, Goyache J, Domenech A, Montana AV, Suarez G, Gomez-Lucia E (2001). Cellular distribution of bovine leukemia virus proteins gp51SU, Pr72(env), and Pr66(gag-pro) in persistently infected cells. Virus Res.

[B117] Katoh I, Yasunaga T, Ikawa Y, Yoshinaka Y (1987). Inhibition of retroviral protease activity by an aspartyl proteinase inhibitor. Nature.

[B118] Zahuczky G, Boross P, Bagossi P, Emri G, Copeland TD, Oroszlan S, Louis JM, Tozser J (2000). Cloning of the bovine leukemia virus proteinase in Escherichia coli and comparison of its specificity to that of human T-cell leukemia virus proteinase. Biochim Biophys Acta.

[B119] Wang H, Norris KM, Mansky LM (2002). Analysis of bovine leukemia virus gag membrane targeting and late domain function. J Virol.

[B120] Wang H, Machesky NJ, Mansky LM (2004). Both the PPPY and PTAP motifs are involved in human T-cell leukemia virus type 1 particle release. J Virol.

[B121] Callahan R, Lieber MM, Todaro GJ, Graves DC, Ferrer JF (1976). Bovine leukemia virus genes in the DNA of leukemic cattle. Science.

[B122] Kettmann R, Portetelle D, Mammerickx M, Cleuter Y, Dekegel D, Galoux M, Ghysdael J, Burny A, Chantrenne H (1976). Bovine leukemia virus: an exogenous RNA oncogenic virus. Proc Natl Acad Sci U S A.

[B123] Avidan O, Meer ME, Oz I, Hizi A (2002). The processivity and fidelity of DNA synthesis exhibited by the reverse transcriptase of bovine leukemia virus. Eur J Biochem.

[B124] Perach M, Hizi A (1999). Catalytic features of the recombinant reverse transcriptase of bovine leukemia virus expressed in bacteria. Virology.

[B125] Mansky LM, Temin HM (1994). Lower mutation rate of bovine leukemia virus relative to that of spleen necrosis virus. J Virol.

[B126] Kashmiri SV, Mehdi R, Ferrer JF (1984). Molecular cloning of covalently closed circular DNA of bovine leukemia virus. J Virol.

[B127] Kettmann R, Couez D, Burny A (1981). Restriction endonuclease mapping of linear unintegrated proviral DNA of bovine leukemia virus. J Virol.

[B128] Reyes RA, Cockerell GL (1996). Unintegrated bovine leukemia virus DNA: association with viral expression and disease. J Virol.

[B129] Tanaka AS, Tanaka M, Komuro K (1998). A highly efficient method for the site-specific integration of transfected plasmids into the genome of mammalian cells using purified retroviral integrase. Gene.

[B130] Tanaka AS, Komuro K (2005). Targeted rearrangement of a chromosomal repeat sequence by transfection of a homologous DNA sequence using purified integrase. Gene Ther.

[B131] Derse D, Diniak AJ, Casey JW, Deininger PL (1985). Nucleotide sequence and structure of integrated bovine leukemia virus long terminal repeats. Virology.

[B132] Coulston J, Naif H, Brandon R, Kumar S, Khan S, Daniel RC, Lavin MF (1990). Molecular cloning and sequencing of an Australian isolate of proviral bovine leukaemia virus DNA: comparison with other isolates. J Gen Virol.

[B133] Rice NR, Stephens RM, Gilden RV (1987). Sequence analysis of the bovine leukemia virus genome. Enzootic bovine leukosis and bovine leukemia virus Martinus Nijhoff Publishing, The Hague, The Netherlands.

[B134] Deshayes L, Levy D, Parodi AL, Levy JP (1980). Spontaneous immune response of bovine leukemia-virus-infected cattle against five different viral proteins. Int J Cancer.

[B135] Zarkik S, Decroly E, Wattiez R, Seidah NG, Burny A, Ruysschaert JM (1997). Comparative processing of bovine leukemia virus envelope glycoprotein gp72 by subtilisin/kexin-like mammalian convertases. FEBS Lett.

[B136] Bruck C, Rensonnet N, Portetelle D, Cleuter Y, Mammerickx M, Burny A, Mamoun R, Guillemain B, Van der Maaten MJ, Ghysdael J (1984). Biologically active epitopes of bovine leukemia virus glycoprotein gp51: their dependence on protein glycosylation and genetic variability. Virology.

[B137] Phillips M, Miller JM, Van der Maaten MJ (1978). Isolation of a precipitating glycoprotein antigen from cell cultures persistently infected with bovine leukemia virus. J Natl Cancer Inst.

[B138] Portetelle D, Bruck C, Mammerickx M, Burny A (1980). In animals infected by bovine leukemia virus (BLV) antibodies to envelope glycoprotein gp51 are directed against the carbohydrate moiety. Virology.

[B139] Schultz AM, Copeland TD, Oroszlan S (1984). The envelope proteins of bovine leukemia virus: purification and sequence analysis. Virology.

[B140] Johnston ER, Radke K (2000). The SU and TM envelope protein subunits of bovine leukemia virus are linked by disulfide bonds, both in cells and in virions. J Virol.

[B141] Bruck C, Mathot S, Portetelle D, Berte C, Franssen JD, Herion P, Burny A (1982). Monoclonal antibodies define eight independent antigenic regions on the bovine leukemia virus (BLV) envelope glycoprotein gp51. Virology.

[B142] Callebaut I, Mornon JP, Burny A, Portetelle D (1994). The bovine leukemia virus (BLV) envelope glycoprotein gp51 as a general model for the design of a subunit vaccine against retroviral infection: mapping of functional sites through immunological and structural data. Leukemia.

[B143] Callebaut I, Voneche V, Mager A, Fumiere O, Krchnak V, Merza M, Zavada J, Mammerickx M, Burny A, Portetelle D (1993). Mapping of B-neutralizing and T-helper cell epitopes on the bovine leukemia virus external glycoprotein gp51. J Virol.

[B144] Portetelle D, Dandoy C, Burny A, Zavada J, Siakkou H, Gras-Masse H, Drobecq H, Tartar A (1989). Synthetic peptides approach to identification of epitopes on bovine leukemia virus envelope glycoprotein gp51. Virology.

[B145] Gatei MH, Good MF, Daniel RC, Lavin MF (1993). T-cell responses to highly conserved CD4 and CD8 epitopes on the outer membrane protein of bovine leukemia virus: relevance to vaccine development. J Virol.

[B146] Hislop AD, Good MF, Mateo L, Gardner J, Gatei MH, Daniel RC, Meyers BV, Lavin MF, Suhrbier A (1998). Vaccine-induced cytotoxic T lymphocytes protect against retroviral challenge. Nat Med.

[B147] Mateo L, Gardner J, Suhrbier A (2001). Delayed emergence of bovine leukemia virus after vaccination with a protective cytotoxic T cell-based vaccine. AIDS Res Hum Retroviruses.

[B148] Johnston ER, Albritton LM, Radke K (2002). Envelope proteins containing single amino acid substitutions support a structural model of the receptor-binding domain of bovine leukemia virus surface protein. J Virol.

[B149] Ghez D, Lepelletier Y, Lambert S, Fourneau JM, Blot V, Janvier S, Arnulf B, van Endert PM, Heveker N, Pique C, Hermine O (2006). Neuropilin-1 is involved in human T-cell lymphotropic virus type 1 entry. J Virol.

[B150] Manel N, Kim FJ, Kinet S, Taylor N, Sitbon M, Battini JL (2003). The ubiquitous glucose transporter GLUT-1 is a receptor for HTLV. Cell.

[B151] Ban J, Portetelle D, Altaner C, Horion B, Milan D, Krchnak V, Burny A, Kettmann R (1993). Isolation and characterization of a 2.3-kilobase-pair cDNA fragment encoding the binding domain of the bovine leukemia virus cell receptor. J Virol.

[B152] Suzuki T, Matsubara Y, Kitani H, Ikeda H (2003). Evaluation of the delta subunit of bovine adaptor protein complex 3 as a receptor for bovine leukaemia virus. J Gen Virol.

[B153] Gatot JS, Callebaut I, Mornon JP, Portetelle D, Burny A, Kerkhofs P, Kettmann R, Willems L (1998). Conservative mutations in the immunosuppressive region of the bovine leukemia virus transmembrane protein affect fusion but not infectivity in vivo. J Biol Chem.

[B154] Johnston ER, Powers MA, Kidd LC, Radke K (1996). Peripheral blood mononuclear cells from sheep infected with a variant of bovine leukemia virus synthesize envelope glycoproteins but fail to induce syncytia in culture. J Virol.

[B155] Voneche V, Portetelle D, Kettmann R, Willems L, Limbach K, Paoletti E, Ruysschaert JM, Burny A, Brasseur R (1992). Fusogenic segments of bovine leukemia virus and simian immunodeficiency virus are interchangeable and mediate fusion by means of oblique insertion in the lipid bilayer of their target cells. Proc Natl Acad Sci U S A.

[B156] Voneche V, Callebaut I, Kettmann R, Brasseur R, Burny A, Portetelle D (1992). The 19-27 amino acid segment of gp51 adopts an amphiphilic structure and plays a key role in the fusion events induced by bovine leukemia virus. J Biol Chem.

[B157] Gatot JS, Callebaut I, Van Lint C, Demonte D, Kerkhofs P, Portetelle D, Burny A, Willems L, Kettmann R (2002). Bovine leukemia virus SU protein interacts with zinc, and mutations within two interacting regions differently affect viral fusion and infectivity in vivo. J Virol.

[B158] Alber G, Kim KM, Weiser P, Riesterer C, Carsetti R, Reth M (1993). Molecular mimicry of the antigen receptor signalling motif by transmembrane proteins of the Epstein-Barr virus and the bovine leukaemia virus. Curr Biol.

[B159] Beaufils P, Choquet D, Mamoun RZ, Malissen B (1993). The (YXXL/I)2 signalling motif found in the cytoplasmic segments of the bovine leukaemia virus envelope protein and Epstein-Barr virus latent membrane protein 2A can elicit early and late lymphocyte activation events. EMBO J.

[B160] Inabe K, Nishizawa M, Tajima S, Ikuta K, Aida Y (1999). The YXXL sequences of a transmembrane protein of bovine leukemia virus are required for viral entry and incorporation of viral envelope protein into virions. J Virol.

[B161] Willems L, Gatot JS, Mammerickx M, Portetelle D, Burny A, Kerkhofs P, Kettmann R (1995). The YXXL signalling motifs of the bovine leukemia virus transmembrane protein are required for in vivo infection and maintenance of high viral loads. J Virol.

[B162] Grange MP, Blot V, Delamarre L, Bouchaert I, Rocca A, Dautry-Varsat A, Dokhelar MC (2000). Identification of two intracellular mechanisms leading to reduced expression of oncoretrovirus envelope glycoproteins at the cell surface. J Virol.

[B163] Novakovic S, Sawai ET, Radke K (2004). Dileucine and YXXL motifs in the cytoplasmic tail of the bovine leukemia virus transmembrane envelope protein affect protein expression on the cell surface. J Virol.

[B164] Hamilton VT, Stone DM, Pritchard SM, Cantor GH (2002). Bovine leukemia virus gp30 transmembrane (TM) protein is not tyrosine phosphorylated: examining potential interactions with host tyrosine-mediated signaling. Virus Res.

[B165] Reichert M, Winnicka A, Willems L, Kettmann R, Cantor GH (2001). Role of the proline-rich motif of bovine leukemia virus transmembrane protein gp30 in viral load and pathogenicity in sheep. J Virol.

[B166] Cantor GH, Pritchard SM, Orlik O, Splitter GA, Davis WC, Reeves R (1999). Bovine leukemia virus transmembrane protein gp30 physically associates with the down-regulatory phosphatase SHP-1. Cell Immunol.

[B167] Ciminale V, Pavlakis GN, Derse D, Cunningham CP, Felber BK (1992). Complex splicing in the human T-cell leukemia virus (HTLV) family of retroviruses: novel mRNAs and proteins produced by HTLV type I. J Virol.

[B168] Lefebvre L, Ciminale V, Vanderplasschen A, D'Agostino D, Burny A, Willems L, Kettmann R (2002). Subcellular localization of the bovine leukemia virus R3 and G4 accessory proteins. J Virol.

[B169] Kerkhofs P, Heremans H, Burny A, Kettmann R, Willems L (1998). In vitro and in vivo oncogenic potential of bovine leukemia virus G4 protein. J Virol.

[B170] Lefebvre L, Vanderplasschen A, Ciminale V, Heremans H, Dangoisse O, Jauniaux JC, Toussaint JF, Zelnik V, Burny A, Kettmann R, Willems L (2002). Oncoviral bovine leukemia virus G4 and human T-cell leukemia virus type 1 p13(II) accessory proteins interact with farnesyl pyrophosphate synthetase. Journal of Virology.

[B171] Willems L, Kerkhofs P, Dequiedt F, Portetelle D, Mammerickx M, Burny A, Kettmann R (1994). Attenuation of bovine leukemia virus by deletion of R3 and G4 open reading frames. Proc Natl Acad Sci U S A.

[B172] Powers MA, Grossman D, Kidd LC, Radke K (1991). Episodic occurrence of antibodies against the bovine leukemia virus Rex protein during the course of infection in sheep. J Virol.

[B173] Rice NR, Simek SL, Dubois GC, Showalter SD, Gilden RV, Stephens RM (1987). Expression of the bovine leukemia virus X region in virus-infected cells. J Virol.

[B174] McGirr KM, Buehring GC (2005). tax and rex Sequences of bovine leukaemia virus from globally diverse isolates: rex amino acid sequence more variable than tax. J Vet Med B Infect Dis Vet Public Health.

[B175] Choi EA, Hope TJ (2005). Mutational analysis of bovine leukemia virus Rex: identification of a dominant-negative inhibitor. J Virol.

[B176] Felber BK, Derse D, Athanassopoulos A, Campbell M, Pavlakis GN (1989). Cross-activation of the Rex proteins of HTLV-I and BLV and of the Rev protein of HIV-1 and nonreciprocal interactions with their RNA responsive elements. New Biol.

[B177] Sagata N, Tsuzuku-Kawamura J, Nagayoshi-Aida M, Shimizu F, Imagawa K, Ikawa Y (1985). Identification and some biochemical properties of the major XBL gene product of bovine leukemia virus. Proc Natl Acad Sci U S A.

[B178] Powers MA, Radke K (1992). Activation of bovine leukemia virus transcription in lymphocytes from infected sheep: rapid transition through early to late gene expression. J Virol.

[B179] Haas L, Divers T, Casey JW (1992). Bovine leukemia virus gene expression in vivo. J Virol.

[B180] Willems L, Bruck C, Portetelle D, Burny A, Kettmann R (1987). Expression of a cDNA clone corresponding to the long open reading frame (XBL-I) of the bovine leukemia virus. Virology.

[B181] Sakakibara N, Kabeya H, Ohashi K, Sugimoto C, Onuma M (1998). Epitope mapping of bovine leukemia virus transactivator protein tax. J Vet Med Sci.

[B182] Ogawa Y, Sagata N, Tsuzuku-Kawamura J, Koyama H, Onuma M, Izawa H, Ikawa Y (1987). Structure of a defective provirus of bovine leukemia virus. Microbiol Immunol.

[B183] Van den BA, Cleuter Y, Chen G, Portetelle D, Mammerickx M, Zagury D, Fouchard M, Coulombel L, Kettmann R, Burny A (1988). Even transcriptionally competent proviruses are silent in bovine leukemia virus-induced sheep tumor cells. Proc Natl Acad Sci U S A.

[B184] Tajima S, Ikawa Y, Aida Y (1998). Complete bovine leukemia virus (BLV) provirus is conserved in BLV-infected cattle throughout the course of B-cell lymphosarcoma development. J Virol.

[B185] Van den BA, Bagnis C, Ciesiolka M, Cleuter Y, Gelderblom H, Kerkhofs P, Griebel P, Mannoni P, Burny A (1999). In vivo rescue of a silent tax-deficient bovine leukemia virus from a tumor-derived ovine B-cell line by recombination with a retrovirally transduced wild-type tax gene. J Virol.

[B186] Twizere JC, Kruys V, Lefebvre L, Vanderplasschen A, Collete D, Debacq C, Lai WS, Jauniaux JC, Bernstein LR, Semmes OJ, Burny A, Blackshear PJ, Kettmann R, Willems L (2003). Interaction of retroviral Tax oncoproteins with tristetraprolin and regulation of tumor necrosis factor-alpha expression. J Natl Cancer Inst.

[B187] Willems L, Kettmann R, Burny A (1991). The amino acid (157-197) peptide segment of bovine leukemia virus p34tax encompass a leucine-rich globally neutral activation domain. Oncogene.

[B188] Chen G, Willems L, Portetelle D, Willard-Gallo KE, Burny A, Gheysen D, Kettmann R (1989). Synthesis of functional bovine leukemia virus (BLV) p34tax protein by recombinant baculoviruses. Virology.

[B189] Derse D (1987). Bovine leukemia virus transcription is controlled by a virus-encoded trans-acting factor and by cis-acting response elements. J Virol.

[B190] Willems L, Gegonne A, Chen G, Burny A, Kettmann R, Ghysdael J (1987). The bovine leukemia virus p34 is a transactivator protein. EMBO J.

[B191] Sakurai M, Taneda A, Nagoya H, Sekikawa K (1991). Construction and functional characterization of mutants of the bovine leukaemia virus trans-activator protein p34tax. J Gen Virol.

[B192] Willems L, Chen G, Portetelle D, Mamoun R, Burny A, Kettmann R (1989). Structural and functional characterization of mutants of the bovine leukemia virus transactivator protein p34. Virology.

[B193] Willems L, Grimonpont C, Heremans H, Rebeyrotte N, Chen G, Portetelle D, Burny A, Kettmann R (1992). Mutations in the bovine leukemia virus Tax protein can abrogate the long terminal repeat-directed transactivating activity without concomitant loss of transforming potential. Proc Natl Acad Sci U S A.

[B194] Tajima S, Aida Y (2000). The region between amino acids 245 and 265 of the bovine leukemia virus (BLV) tax protein restricts transactivation not only via the BLV enhancer but also via other retrovirus enhancers. J Virol.

[B195] Tajima S, Aida Y (2002). Mutant tax protein from bovine leukemia virus with enhanced ability to activate the expression of c-fos. J Virol.

[B196] Tajima S, Takahashi M, Takeshima SN, Konnai S, Yin SA, Watarai S, Tanaka Y, Onuma M, Okada K, Aida Y (2003). A mutant form of the tax protein of bovine leukemia virus (BLV), with enhanced transactivation activity, increases expression and propagation of BLV in vitro but not in vivo. J Virol.

[B197] Takahashi M, Tajima S, Okada K, Davis WC, Aida Y (2005). Involvement of bovine leukemia virus in induction and inhibition of apoptosis. Microbes Infect.

[B198] Willems L, Heremans H, Chen G, Portetelle D, Billiau A, Burny A, Kettmann R (1990). Cooperation between bovine leukaemia virus transactivator protein and Ha-ras oncogene product in cellular transformation. EMBO J.

[B199] Willems L, Grimonpont C, Kerkhofs P, Capiau C, Gheysen D, Conrath K, Roussef R, Mamoun R, Portetelle D, Burny A, Adam E, Lefebvre L, Twizere JC, Heremans H, Kettmann R (1998). Phosphorylation of bovine leukemia virus Tax protein is required for in vitro transformation but not for transactivation. Oncogene.

[B200] Twizere JC, Kerkhofs P, Burny A, Portetelle D, Kettmann R, Willems L (2000). Discordance between bovine leukemia virus tax immortalization in vitro and oncogenicity in vivo. J Virol.

[B201] Szynal M, Cleuter Y, Beskorwayne T, Bagnis C, Van Lint C, Kerkhofs P, Burny A, Martiat P, Griebel P, Van den BA (2003). Disruption of B-cell homeostatic control mediated by the BLV-Tax oncoprotein: association with the upregulation of Bcl-2 and signaling through NF-kappaB. Oncogene.

[B202] Klener P, Szynal M, Cleuter Y, Merimi M, Duvillier H, Lallemand F, Bagnis C, Griebel P, Sotiriou C, Burny A, Martiat P, Van den BA (2006). Insights into gene expression changes impacting B-cell transformation: cross-species microarray analysis of bovine leukemia virus tax-responsive genes in ovine B cells. J Virol.

[B203] Philpott SM, Buehring GC (1999). Defective DNA repair in cells with human T-cell leukemia/bovine leukemia viruses: role of tax gene. J Natl Cancer Inst.

[B204] Twizere JC, Lefebvre L, Collete D, Debacq C, Urbain P, Heremans H, Jauniaux JC, Burny A, Willems L, Kettmann R (2005). The homeobox protein MSX2 interacts with tax oncoproteins and represses their transactivation activity. J Biol Chem.

[B205] Twizere JC, Springael JY, Boxus M, Burny A, Dequiedt F, Dewulf JF, Duchateau J, Portetelle D, Urbain P, Van Lint C, Green PL, Mahieux R, Parmentier M, Willems L, Kettmann R (2006). Human T-cell leukemia virus type-1 Tax oncoprotein regulates G protein signaling. Blood.

[B206] Buehring GC, Kramme PM, Schultz RD (1994). Evidence for bovine leukemia virus in mammary epithelial cells of infected cows. Lab Invest.

[B207] Domenech A, Goyache J, Llames L, Jesus PM, Suarez G, Gomez-Lucia E (2000). In vitro infection of cells of the monocytic/macrophage lineage with bovine leukaemia virus. J Gen Virol.

[B208] Fulton BE, Portella M, Radke K (2006). Dissemination of bovine leukemia virus-infected cells from a newly infected sheep lymph node. J Virol.

[B209] Heeney JL, Valli PJ, Jacobs RM, Valli VE (1992). Evidence for bovine leukemia virus infection of peripheral blood monocytes and limited antigen expression in bovine lymphoid tissue. Lab Invest.

[B210] Rovnak J, Casey JW, Boyd AL, Gonda MA, Cockerell GL (1991). Isolation of bovine leukemia virus infected endothelial cells from cattle with persistent lymphocytosis. Lab Invest.

[B211] Stott ML, Thurmond MC, Dunn SJ, Osburn BI, Stott JL (1991). Integrated bovine leukosis proviral DNA in T helper and T cytotoxic/suppressor lymphocytes. J Gen Virol.

[B212] Altreuther G, Llames L, Neuenschwander S, Langhans W, Werling D (2001). Morphologic and functional changes in bovine monocytes infected in vitro with the bovine leukaemia virus. Scand J Immunol.

[B213] Wu D, Murakami K, Morooka A, Jin H, Inoshima Y, Sentsui H (2003). In vivo transcription of bovine leukemia virus and bovine immunodeficiency-like virus. Virus Res.

[B214] Aida Y, Miyasaka M, Okada K, Onuma M, Kogure S, Suzuki M, Minoprio P, Levy D, Ikawa Y (1989). Further phenotypic characterization of target cells for bovine leukemia virus experimental infection in sheep. Am J Vet Res.

[B215] Aida Y, Okada K, Amanuma H (1993). Phenotype and ontogeny of cells carrying a tumor-associated antigen that is expressed on bovine leukemia virus-induced lymphosarcoma. Cancer Res.

[B216] Mirsky ML, Da Y, Lewin HA (1993). Detection of bovine leukemia virus proviral DNA in individual cells. PCR Methods Appl.

[B217] Mirsky ML, Olmstead CA, Da Y, Lewin HA (1996). The prevalence of proviral bovine leukemia virus in peripheral blood mononuclear cells at two subclinical stages of infection. J Virol.

[B218] Vernau W, Jacobs RM, Valli VE, Heeney JL (1997). The immunophenotypic characterization of bovine lymphomas. Vet Pathol.

[B219] Wu D, Takahashi K, Murakami K, Tani K, Koguchi A, Asahina M, Goryo M, Aida Y, Okada K (1996). B-1a, B-1b and conventional B cell lymphoma from enzootic bovine leukosis. Vet Immunol Immunopathol.

[B220] Saini SS, Allore B, Jacobs RM, Kaushik A (1999). Exceptionally long CDR3H region with multiple cysteine residues in functional bovine IgM antibodies. Eur J Immunol.

[B221] Yan XJ, Albesiano E, Zanesi N, Yancopoulos S, Sawyer A, Romano E, Petlickovski A, Efremov DG, Croce CM, Chiorazzi N (2006). B cell receptors in TCL1 transgenic mice resemble those of aggressive, treatment-resistant human chronic lymphocytic leukemia. Proc Natl Acad Sci U S A.

[B222] Depelchin A, Letesson JJ, Lostrie-Trussart N, Mammerickx M, Portetelle D, Burny A (1989). Bovine leukemia virus (BLV)-infected B-cells express a marker similar to the CD5 T cell marker. Immunol Lett.

[B223] Letesson JJ, Mager A, Mammerickx M, Burny A, Depelchin A (1990). B cells from bovine leukemia virus- (BLV) infected sheep with hematological disorders express the CD5 T cell marker. Leukemia.

[B224] Matheise JP, Delcommenne M, Mager A, Didembourg CH, Letesson JJ (1992). CD5+ B cells from bovine leukemia virus infected cows are activated cycling cells responsive to interleukin 2. Leukemia.

[B225] Meirom R, Moss S, Brenner J (1997). Bovine leukemia virus-gp51 antigen expression is associated with CD5 and IgM markers on infected lymphocytes. Vet Immunol Immunopathol.

[B226] Cantor GH, Palmer GH (1992). Antisense oligonucleotide inhibition of bovine leukemia virus tax expression in a cell-free system. Antisense Res Dev.

[B227] Hamilton VT, Stone DM, Cantor GH (2003). Translocation of the B cell receptor to lipid rafts is inhibited in B cells from BLV-infected, persistent lymphocytosis cattle. Virology.

[B228] Chevallier N, Berthelemy M, Le Rhun D, Laine V, Levy D, Schwartz-Cornil I (1998). Bovine leukemia virus-induced lymphocytosis and increased cell survival mainly involve the CD11b+ B-lymphocyte subset in sheep. J Virol.

[B229] Murakami K, Okada K, Ikawa Y, Aida Y (1994). Bovine leukemia virus induces CD5- B cell lymphoma in sheep despite temporarily increasing CD5+ B cells in asymptomatic stage. Virology.

[B230] Takahashi M, Tajima S, Takeshima SN, Konnai S, Yin SA, Okada K, Davis WC, Aida Y (2004). Ex vivo survival of peripheral blood mononuclear cells in sheep induced by bovine leukemia virus (BLV) mainly occurs in CD5- B cells that express BLV. Microbes Infect.

[B231] Aida Y, Okada K, Ohtsuka M, Amanuma H (1992). Tumor-associated M(r) 34,000 and M(r) 32,000 membrane glycoproteins that are serine phosphorylated specifically in bovine leukemia virus-induced lymphosarcoma cells. Cancer Res.

[B232] Aida Y, Amanuma H, Okada K (1994). Identification of tumor-associated antigen that is expressed on bovine leukemia virus-induced lymphosarcoma cells and expression of its human homologue in human T-cell lymphotrophic virus I-infected cell lines. Leukemia.

[B233] Aida Y, Nishino Y, Amanuma H, Murakami K, Okada K, Ikawa Y (1997). The role of tumor-associated antigen in bovine leukemia virus-induced lymphosarcoma. Leukemia.

[B234] Stone DM, Hof AJ, Davis WC (1995). Up-regulation of IL-2 receptor alpha and MHC class II expression on lymphocyte subpopulations from bovine leukemia virus infected lymphocytotic cows. Vet Immunol Immunopathol.

[B235] Hanon E, Stinchcombe JC, Saito M, Asquith BE, Taylor GP, Tanaka Y, Weber JN, Griffiths GM, Bangham CR (2000). Fratricide among CD8(+) T lymphocytes naturally infected with human T cell lymphotropic virus type I. Immunity.

[B236] Nagai M, Brennan MB, Sakai JA, Mora CA, Jacobson S (2001). CD8(+) T cells are an in vivo reservoir for human T-cell lymphotropic virus type I. Blood.

[B237] Willems L, Portetelle D, Kerkhofs P, Chen G, Burny A, Mammerickx M, Kettmann R (1992). In vivo transfection of bovine leukemia provirus into sheep. Virology.

[B238] Rovnak J, Boyd AL, Casey JW, Gonda MA, Jensen WA, Cockerell GL (1993). Pathogenicity of molecularly cloned bovine leukemia virus. J Virol.

[B239] Bartoe JT, Albrecht B, Collins ND, Robek MD, Ratner L, Green PL, Lairmore MD (2000). Functional role of pX open reading frame II of human T-lymphotropic virus type 1 in maintenance of viral loads in vivo. J Virol.

[B240] Collins ND, Newbound GC, Albrecht B, Beard JL, Ratner L, Lairmore MD (1998). Selective ablation of human T-cell lymphotropic virus type 1 p12I reduces viral infectivity in vivo. Blood.

[B241] Silverman LR, Phipps AJ, Montgomery A, Ratner L, Lairmore MD (2004). Human T-cell lymphotropic virus type 1 open reading frame II-encoded p30II is required for in vivo replication: evidence of in vivo reversion. J Virol.

[B242] Van den BA, Cleuter Y, Beskorwayne T, Kerkhofs P, Szynal M, Bagnis C, Burny A, Griebel P (2001). CD154 costimulated ovine primary B cells, a cell culture system that supports productive infection by bovine leukemia virus. J Virol.

[B243] Kettmann R, Bruck C, Mammerickx M, Burny A (1980). BLV proviral DNA in the genome of the target lymphocyte. Arch Geschwulstforsch.

[B244] Debacq C, Sanchez Alcaraz MT, Mortreux F, Kerkhofs P, Kettmann R, Willems L (2004). Reduced proviral loads during primo-infection of sheep by Bovine Leukemia virus attenuated mutants. Retrovirology.

[B245] Moules V, Pomier C, Sibon D, Gabet AS, Reichert N, Kerkhofs P, Willems L, Mortreux F, Wattel E (2005). Fate of premalignant clones during the asymptomatic phase preceding lymphoid malignancy. Cancer Research.

[B246] Kettmann R, Deschamps J, Cleuter Y, Couez D, Burny A, Marbaix G (1982). Leukemogenesis by bovine leukemia virus: proviral DNA integration and lack of RNA expression of viral long terminal repeat and 3' proximate cellular sequences. Proc Natl Acad Sci U S A.

[B247] Deschamps J, Kettmann R, Burny A (1981). Experiments with cloned complete tumor-derived bovine leukemia virus information prove that the virus is totally exogenous to its target animal species. J Virol.

[B248] Ogawa Y, Sagata N, Tsuzuku-Kawamura J, Onuma M, Izawa H, Ikawa Y (1986). No involvement of bovine leukemia virus in sporadic bovine lymphosarcoma. Microbiol Immunol.

[B249] Gregoire D, Couez D, Deschamps J, Heuertz S, Hors-Cayla MC, Szpirer J, Szpirer C, Burny A, Huez G, Kettmann R (1984). Different bovine leukemia virus-induced tumors harbor the provirus in different chromosomes. J Virol.

[B250] Kettmann R, Deschamps J, Couez D, Claustriaux JJ, Palm R, Burny A (1983). Chromosome integration domain for bovine leukemia provirus in tumors. J Virol.

[B251] Kettmann R, Marbaix G, Cleuter Y, Portetelle D, Mammerickx M, Burny A (1980). Genomic integration of bovine leukemia provirus and lack of viral RNA expression in the target cells of cattle with different responses to BLV infection. Leuk Res.

[B252] Kettmann R, Cleuter Y, Gregoire D, Burny A (1985). Role of the 3' long open reading frame region of bovine leukemia virus in the maintenance of cell transformation. J Virol.

[B253] Gaynor EM, Mirsky ML, Lewin HA (1996). Use of flow cytometry and RT-PCR for detecting gene expression by single cells. Biotechniques.

[B254] Jensen WA, Rovnak J, Cockerell GL (1991). In vivo transcription of the bovine leukemia virus tax/rex region in normal and neoplastic lymphocytes of cattle and sheep. J Virol.

[B255] Lagarias DM, Radke K (1989). Transcriptional activation of bovine leukemia virus in blood cells from experimentally infected, asymptomatic sheep with latent infections. J Virol.

[B256] Radke K, Sigala TJ, Grossman D (1992). Transcription of bovine leukemia virus in peripheral blood cells obtained during early infection in vivo. Microb Pathog.

[B257] Gupta P, Ferrer JF (1982). Expression of bovine leukemia virus genome is blocked by a nonimmunoglobulin protein in plasma from infected cattle. Science.

[B258] Gupta P, Kashmiri SV, Ferrer JF (1984). Transcriptional control of the bovine leukemia virus genome: role and characterization of a non-immunoglobulin plasma protein from bovine leukemia virus-infected cattle. J Virol.

[B259] van den Heuvel MJ, Jefferson BJ, Jacobs RM (2005). Isolation of a bovine plasma fibronectin-containing complex which inhibits the expression of bovine leukemia virus p24. J Virol.

[B260] Tsukiyama K, Onuma M, Izawa H (1987). Effect of platelet-derived factor on expression of bovine leukemia virus genome. Arch Virol.

[B261] Tajima S, Aida Y (2005). Induction of expression of bovine leukemia virus (BLV) in blood taken from BLV-infected cows without removal of plasma. Microbes Infect.

[B262] Driscoll DM, Baumgartener LE, Olson C (1977). Concanavalin A and the production of bovine leukemia virus antigen in short-term lymphocyte cultures. J Natl Cancer Inst.

[B263] Trueblood ES, Brown WC, Palmer GH, Davis WC, Stone DM, McElwain TF (1998). B-lymphocyte proliferation during bovine leukemia virus-induced persistent lymphocytosis is enhanced by T-lymphocyte-derived interleukin-2. J Virol.

[B264] Stock ND, Ferrer JF (1972). Replicating C-type virus in phytohemagglutinin-treated buffy-coat cultures of bovine origin. J Natl Cancer Inst.

[B265] Kidd LC, Radke K (1996). Lymphocyte activators elicit bovine leukemia virus expression differently as asymptomatic infection progresses. Virology.

[B266] Jensen WA, Wicks-Beard BJ, Cockerell GL (1992). Inhibition of protein kinase C results in decreased expression of bovine leukemia virus. J Virol.

[B267] Kerkhofs P, Adam E, Droogmans L, Portetelle D, Mammerickx M, Burny A, Kettmann R, Willems L (1996). Cellular pathways involved in the ex vivo expression of bovine leukemia virus. J Virol.

[B268] Cornil I, Delon P, Parodi AL, Levy D (1988). T-B cell cooperation for bovine leukemia virus expression in ovine lymphocytes. Leukemia.

[B269] Djilali S, Parodi AL, Levy D (1987). Bovine leukemia virus replicates in sheep B lymphocytes under a T cell released factor. Eur J Cancer Clin Oncol.

[B270] Pyeon D, O'Reilly KL, Splitter GA (1996). Increased interleukin-10 mRNA expression in tumor-bearing or persistently lymphocytotic animals infected with bovine leukemia virus. J Virol.

[B271] Yakobson B, Brenner J, Ungar-Waron H, Trainin Z (1998). Short-termed expression of interleukin-12 during experimental BLV infection may direct disease progression to persistent lymphocytosis. Vet Immunol Immunopathol.

[B272] Pyeon D, Splitter GA (1999). Regulation of bovine leukemia virus tax and pol mRNA levels by interleukin-2 and -10. J Virol.

[B273] Pyeon D, Diaz FJ, Splitter GA (2000). Prostaglandin E(2) increases bovine leukemia virus tax and pol mRNA levels via cyclooxygenase 2: regulation by interleukin-2, interleukin-10, and bovine leukemia virus. J Virol.

[B274] Amills M, Ramiya V, Norimine J, Olmstead CA, Lewin HA (2002). Reduced IL-2 and IL-4 mRNA expression in CD4+ T cells from bovine leukemia virus-infected cows with persistent lymphocytosis. Virology.

[B275] Isaacson JA, Flaming KP, Roth JA (1998). Increased MHC class II and CD25 expression on lymphocytes in the absence of persistent lymphocytosis in cattle experimentally infected with bovine leukemia virus. Vet Immunol Immunopathol.

[B276] Sordillo LM, Hicks CR, Pighetti GM (1994). Altered interleukin-2 production by lymphocyte populations from bovine leukemia virus-infected cattle. Proc Soc Exp Biol Med.

[B277] Stone DM, McElwain TF, Davis WC (1994). Enhanced B-lymphocyte expression of IL-2R alpha associated with T lymphocytosis in BLV-infected persistently lymphocytotic cows. Leukemia.

[B278] Keefe RG, Ferrick DA, Stott JL (1997). Cytokine transcription in lymph nodes of cattle in different stages of bovine leukemia virus infection. Vet Immunol Immunopathol.

[B279] Amills M, Norimine J, Olmstead CA, Lewin HA (2004). Cytokine mRNA expression in B cells from bovine leukemia virus-infected cattle with persistent lymphocytosis. Cytokine.

[B280] Meirom R, Moss S, Brenner J, Heller D, Trainin Z (1997). Levels and role of cytokines in bovine leukemia virus (BLV) infection. Leukemia.

[B281] Keefe RG, Choi Y, Ferrick DA, Stott JL (1997). Bovine cytokine expression during different phases of bovine leukemia virus infection. Vet Immunol Immunopathol.

[B282] Konnai S, Usui T, Ohashi K, Onuma M (2003). The rapid quantitative analysis of bovine cytokine genes by real-time RT-PCR. Vet Microbiol.

[B283] Muller C, Coffey TJ, Koss M, Teifke JP, Langhans W, Werling D (2003). Lack of TNF alpha supports persistence of a plasmid encoding the bovine leukaemia virus in TNF(-/-) mice. Vet Immunol Immunopathol.

[B284] Kabeya H, Ohashi K, Onuma M (2001). Host immune responses in the course of bovine leukemia virus infection. J Vet Med Sci.

[B285] Usui T, Konnai S, Ohashi K, Onuma M (2006). Expression of tumor necrosis factor-alpha in IgM+ B-cells from bovine leukemia virus-infected lymphocytotic sheep. Vet Immunol Immunopathol.

[B286] Kabeya H, Ohashi K, Oyunbileg N, Nagaoka Y, Aida Y, Sugimoto C, Yokomizo Y, Onuma M (1999). Up-regulation of tumor necrosis factor alpha mRNA is associated with bovine-leukemia virus (BLV) elimination in the early phase of infection. Vet Immunol Immunopathol.

[B287] Konnai S, Usui T, Ikeda M, Kohara J, Hirata T, Okada K, Ohashi K, Onuma M (2006). Tumor necrosis factor-alpha up-regulation in spontaneously proliferating cells derived from bovine leukemia virus-infected cattle. Arch Virol.

[B288] Konnai S, Usui T, Ikeda M, Kohara J, Hirata T, Okada K, Ohashi K, Onuma M (2005). Imbalance of tumor necrosis factor receptors during progression in bovine leukemia virus infection. Virology.

[B289] Sentsui H, Murakami K, Inoshima Y, Yokoyama T, Inumaru S (2001). Anti-viral effect of recombinant bovine interferon gamma on bovine leukaemia virus. Cytokine.

[B290] Usui T, Konnai S, Ohashi K, Onuma M (2006). Interferon-gamma expression associated with suppression of bovine leukemia virus at the early phase of infection in sheep. Vet Immunol Immunopathol.

[B291] Murakami K, Sentsui H, Inoshima Y, Inumaru S (2004). Increase in gammadelta T cells in the blood of cattle persistently infected with bovine leukemia virus following administration of recombinant bovine IFN-gamma. Vet Immunol Immunopathol.

[B292] Yakobson B, Brenner J, Ungar-Waron H, Trainin Z (2000). Cellular immune response cytokine expression during the initial stage of bovine leukemia virus (BLV) infection determines the disease progression to persistent lymphocytosis. Comp Immunol Microbiol Infect Dis.

[B293] Klintevall K, Fuxler L, Fossum C (1997). Bovine leukemia virus: early reflections in blood after an experimental infection of calves. Comp Immunol Microbiol Infect Dis.

[B294] Kohara J, Yokomizo Y (2007). In Vitro and In Vivo Effects of Recombinant Bovine Interferon-tau on Bovine Leukemia Virus. J Vet Med Sci.

[B295] Dequiedt F, Kettmann R, Burny A, Willems L (1995). Nucleotide sequence of the ovine P53 tumor-suppressor cDNA and its genomic organization. DNA Seq.

[B296] Ishiguro N, Furuoka H, Matsui T, Horiuchi M, Shinagawa M, Asahina M, Okada K (1997). p53 mutation as a potential cellular factor for tumor development in enzootic bovine leukosis. Vet Immunol Immunopathol.

[B297] Zhuang W, Tajima S, Okada K, Ikawa Y, Aida Y (1997). Point mutation of p53 tumor suppressor gene in bovine leukemia virus-induced lymphosarcoma. Leukemia.

[B298] Tajima S, Zhuang WZ, Kato MV, Okada K, Ikawa Y, Aida Y (1998). Function and Conformation of Wild-Type p53 Protein Are Influenced by Mutations in Bovine Leukemia Virus-Induced B-Cell Lymphosarcoma. Virology.

[B299] Reyes RA, Cockerell GL (1998). Increased ratio of bcl-2/bax expression is associated with bovine leukemia virus-induced leukemogenesis in cattle. Virology.

[B300] Dequiedt F, Kettmann R, Burny A, Willems L (1995). Mutations in the p53 tumor-suppressor gene are frequently associated with bovine leukemia virus-induced leukemogenesis in cattle but not in sheep. Virology.

[B301] Schnurr MW, Carter RF, Dube ID, Valli VE, Jacobs RM (1994). Nonrandom chromosomal abnormalities in bovine lymphoma. Leuk Res.

[B302] Lewin HA, Russell GC, Glass EJ (1999). Comparative organization and function of the major histocompatibility complex of domesticated cattle. Immunol Rev.

[B303] Lewin HA, Bernoco D (1986). Evidence for BoLA-linked resistance and susceptibility to subclinical progression of bovine leukaemia virus infection. Anim Genet.

[B304] Lewin HA, Wu MC, Stewart JA, Nolan TJ (1988). Association between BoLA and subclinical bovine leukemia virus infection in a herd of Holstein-Friesian cows. Immunogenetics.

[B305] Xu A, van Eijk MJ, Park C, Lewin HA (1993). Polymorphism in BoLA-DRB3 exon 2 correlates with resistance to persistent lymphocytosis caused by bovine leukemia virus. J Immunol.

[B306] Zanotti M, Poli G, Ponti W, Polli M, Rocchi M, Bolzani E, Longeri M, Russo S, Lewin HA, van Eijk MJ (1996). Association of BoLA class II haplotypes with subclinical progression of bovine leukaemia virus infection in Holstein-Friesian cattle. Anim Genet.

[B307] Mirsky ML, Olmstead C, Da Y, Lewin HA (1998). Reduced bovine leukaemia virus proviral load in genetically resistant cattle. Anim Genet.

[B308] Konnai S, Usui T, Ikeda M, Kohara J, Hirata TI, Okada K, Ohashi K, Onuma M (2006). Tumor necrosis factor-alpha genetic polymorphism may contribute to progression of bovine leukemia virus-infection. Microbes Infect.

[B309] Nagaoka Y, Kabeya H, Onuma M, Kasai N, Okada K, Aida Y (1999). Ovine MHC class II DRB1 alleles associated with resistance or susceptibility to development of bovine leukemia virus-induced ovine lymphoma. Cancer Res.

[B310] Konnai S, Takeshima SN, Tajima S, Yin SA, Okada K, Onuma M, Aida Y (2003). The influence of ovine MHC class II DRB1 alleles on immune response in bovine leukemia virus infection. Microbiol Immunol.

[B311] Portetelle D, Mammerickx M, Burny A (1989). Use of two monoclonal antibodies in an ELISA test for the detection of antibodies to bovine leukaemia virus envelope protein gp51. J Virol Methods.

[B312] Radke K, Grossman D, Kidd LC (1990). Humoral immune response of experimentally infected sheep defines two early periods of bovine leukemia virus replication. Microb Pathog.

[B313] Portetelle D, Bruck C, Burny A, Dekegel D, Mammerickx M, Urbain J (1978). Detection of complement-dependent lytic antibodies in sera from bovine leukemia virus-infected animals. Ann Rech Vet.

[B314] Nagy DW, Tyler JW, Stoker A, Kleiboeker SB (2002). Association between the strength of serologic recognition of bovine leukosis virus and lymphocyte count in bovine leukosis virus-infected cows. J Am Vet Med Assoc.

[B315] Lundberg P, Splitter GA (2000). gammadelta(+) T-Lp6phocyte cytotoxicity against envelope-expressing target cells is unique to the alymphocytic state of bovine leukemia virus infection in the natural host. J Virol.

[B316] Kabeya H, Ohashi K, Sugimoto C, Onuma M (1999). Characterization of immune responses caused by bovine leukemia virus envelope peptides in sheep. J Vet Med Sci.

[B317] Stone DM, Norton LK, Chambers JC, Meek WJ (2000). CD4 T lymphocyte activation in BLV-induced persistent B lymphocytosis in cattle. Clin Immunol.

[B318] Oldstone MBA (2006). Viral persistence: Parameters, mechanisms and future predictions. Virology.

[B319] Brenner J, Van Haam M, Savir D, Trainin Z (1989). The implication of BLV infection in the productivity, reproductive capacity and survival rate of a dairy cow. Vet Immunol Immunopathol.

[B320] Wattel E, Cavrois M, Gessain A, WainHobson S (1996). Clonal expansion of infected cells: A way of life for HTLV-I. Journal of Acquired Immune Deficiency Syndromes and Human Retrovirology.

[B321] Dequiedt F, Hanon E, Kerkhofs P, Pastoret PP, Portetelle D, Burny A, Kettmann R, Willems L (1997). Both wild-type and strongly attenuated bovine leukemia viruses protect peripheral blood mononuclear cells from apoptosis. J Virol.

[B322] Schwartz-Cornil I, Chevallier N, Belloc C, Le Rhun D, Laine V, Berthelemy M, Mateo A, Levy D (1997). Bovine leukaemia virus-induced lymphocytosis in sheep is associated with reduction of spontaneous B cell apoptosis. J Gen Virol.

[B323] Alcaraz TS, Kerkhofs P, Reichert M, Kettmann R, Willems L (2004). Involvement of glutathione as a mechanism of indirect protection against spontaneous ex vivo apoptosis associated with bovine leukemia virus. Journal of Virology.

[B324] Debacq C, Asquith B, Reichert M, Burny A, Kettmann R, Willems L (2003). Reduced cell turnover in bovine leukemia virus-infected, persistently lymphocytotic cattle. J Virol.

[B325] Dequiedt F, Cantor GH, Hamilton VT, Pritchard SM, Davis WC, Kerkhofs P, Burny A, Kettmann R, Willems L (1999). Bovine leukemia virus-induced persistent lymphocytosis in cattle does not correlate with increased ex vivo survival of B lymphocytes. J Virol.

[B326] Gupta P, Kashmiri SV, Erisman MD, Rothberg PG, Astrin SM, Ferrer JF (1986). Enhanced expression of the c-myc gene in bovine leukemia virus-induced bovine tumors. Cancer Res.

[B327] Hailata N, Johnson R, al Bagdadi F, Hanash S (1995). Proliferating cell nuclear antigen expression in sheep infected with bovine leukemia virus. Vet Immunol Immunopathol.

[B328] Stone DM, Norton LK, Magnuson NS, Davis WC (1996). Elevated pim-1 and c-myc proto-oncogene induction in B lymphocytes from BLV-infected cows with persistent B lymphocytosis. Leukemia.

[B329] Debacq C, Asquith B, Kerkhofs P, Portetelle D, Burny A, Kettmann R, Willems L (2002). Increased cell proliferation, but not reduced cell death, induces lymphocytosis in bovine leukemia virus-infected sheep. Proc Natl Acad Sci U S A.

[B330] Jacobs A, Clark RE (1986). Pathogenesis and Clinical Variations in the Myelodysplastic Syndromes. Clinics in Haematology.

[B331] Ristevski B, Young AJ, Dudler L, Cahill RNP, Kimpton W, Washington E, Hay JB (2003). Tracking dendritic cells: use of an in situ method to label all blood leukocytes. International Immunology.

[B332] Hay JB, Andrade WN (1998). Lymphocyte recirculation, exercise, and immune responses. Can J Physiol Pharmacol.

[B333] Hein WR, Griebel PJ (2003). A road less travelled: large animal models in immunological research. Nat Rev Immunol.

[B334] Young AJ, Hay JB, Mackay CR (1993). Lymphocyte recirculation and life span in vivo. Curr Top Microbiol Immunol.

[B335] Debacq C, Gillet N, Asquith B, Sanchez-Alcaraz MT, Florins A, Boxus M, Schwartz-Cornil I, Bonneau M, Jean G, Kerkhofs P, Hay J, Thewis A, Kettmann R, Willems L (2006). Peripheral blood B-cell death compensates for excessive proliferation in lymphoid tissues and maintains homeostasis in bovine leukemia virus-infected sheep. J Virol.

[B336] Asquith B, Debacq C, Florins A, Gillet N, Sanchez Alcaraz MT, Mosley A, Willems L (2006). Quantifying lymphocyte kinetics in vivo using CFSE. Proceedings of the Royal Society of London.

[B337] Florins A, Gillet N, Asquith B, Debacq C, Jean G, Schwartz-Cornil I, Bonneau M, Burny A, Reichert M, Kettmann R, Willems L (2006). Spleen-Dependent Turnover of CD11b Peripheral Blood B Lymphocytes in Bovine Leukemia Virus-Infected Sheep. J Virol.

[B338] Achachi A, Florins A, Gillet N, Debacq C, Urbain P, Foutsop GM, Vandermeers F, Jasik A, Reichert M, Kerkhofs P, Lagneaux L, Burny A, Kettmann R, Willems L (2005). Valproate activates bovine leukemia virus gene expression, triggers apoptosis, and induces leukemia/lymphoma regression in vivo. Proc Natl Acad Sci U S A.

[B339] Kamoi K, Yamamoto K, Misawa A, Miyake A, Ishida T, Tanaka Y, Mochizuki M, Watanabe T (2006). SUV39H1 interacts with HTLV-1 Tax and abrogates Tax transactivation of HTLV-1 LTR. Retrovirology.

[B340] Hivin P, Basbous J, Raymond F, Henaff D, Arpin-Andre C, Robert-Hebmann V, Barbeau B, Mesnard JM (2007). The HBZ-SP1 isoform of human T-cell leukemia virus type I represses JunB activity by sequestration into nuclear bodies. Retrovirology.

[B341] Satou Y, Yasunaga J, Yoshida M, Matsuoka M (2006). HTLV-I basic leucine zipper factor gene mRNA supports proliferation of adult T cell leukemia cells. Proc Natl Acad Sci U S A.

[B342] Inoue J, Seiki M, Taniguchi T, Tsuru S, Yoshida M (1986). Induction of interleukin 2 receptor gene expression by p40x encoded by human T-cell leukemia virus type 1. EMBO J.

[B343] Asquith B, Mosley AJ, Heaps A, Tanaka Y, Taylor GP, McLean AR, Bangham CR (2005). Quantification of the virus-host interaction in human T lymphotropic virus I infection. Retrovirology.

[B344] Lagarias DM, Radke K (1990). Transient increases of blood mononuclear cells that could express bovine leukemia virus early after experimental infection of sheep. Microb Pathog.

[B345] Yoo C, Jones P (2006). Epigenetic therapy of cancer: past, present and future. Nature reviews.

[B346] Blaheta RA, Nau H, Michaelis M, Cinatl J (2002). Valproate and valproate-analogues: Potent tools to fight against cancer. Current Medicinal Chemistry.

[B347] Drummond DC, Noble CO, Kirpotin DB, Guo ZX, Scott GK, Benz CC (2005). Clinical development of histone deacetylase inhibitors as anticancer agents. Annual Review of Pharmacology and Toxicology.

[B348] Insinga A, Monestiroli S, Ronzoni S, Gelmetti V, Marchesi F, Viale A, Altucci L, Nervi C, Minucci S, Pelicci PG (2005). Inhibitors of histone deacetylases induce tumor-selective apoptosis through activation of the death receptor pathway. Nature Medicine.

[B349] Nebbioso A, Clarke N, Voltz E, Germain E, Ambrosino C, Bontempo P, Alvarez R, Schiavone EM, Ferrara F, Bresciani F, Weisz A, de Lera AR, Gronemeyer H, Altucci L (2005). Tumor-selective action of HDAC inhibitors involves TRAIL induction in acute myeloid leukemia cells. Nature Medicine.

[B350] Nakagawa M, Nakahara K, Maruyama Y, Kawabata M, Higuchi I, Kubota H, Izumo S, Arimura K, Osame M (1996). Therapeutic trials in 200 patients with HTLV-I-associated myelopathy/tropical spastic paraparesis. Journal of Neurovirology.

[B351] Oh U, Yamano Y, Mora CA, Ohayon J, Bagnato F, Butman JA, Dambrosia J, Leist TP, McFarland H, Jacobson S (2005). Interferon-beta 1a therapy in human T-lymphotropic virus type I-associated neurologic disease. Annals of Neurology.

[B352] Saito M, Nakagawa M, Kaseda S, Matsuzaki T, Jonosono M, Eiraku N, Kubota R, Takenouchi N, Nagai M, Furukawa Y, Usuku K, Izumo S, Osame M (2004). Decreased human T lymphotropic virus type I (HTLV-I) provirus load and alteration in T cell phenotype after interferon-alpha therapy for HTLV-I-associated myelopathy/tropical spastic paraparesis. Journal of Infectious Diseases.

[B353] Ikegami M, Umehara F, Ikegami N, Maekawa R, Osame M (2002). Selective matrix metalloproteinase inhibitor, N-biphenyl sulfonyl phenylalanine hydroxamic acid, inhibits the migration of CD4(+) T lymphocytes in patients with HTLV-I-associated myelopathy. Journal of Neuroimmunology.

[B354] Machuca A, Soriano V (2000). In vivo fluctuation of HTLV-I and HTLV-II proviral load in patients receiving antiretroviral drugs. Journal of Acquired Immune Deficiency Syndromes.

[B355] Sheremata WA, Benedict D, Squilacote DC, Sazant A, Defreitas E (1993). High-Dose Zidovudine Induction in Htlv-I-Associated Myelopathy - Safety and Possible Efficacy. Neurology.

[B356] Taylor GP, Hall SE, Navarrete S, Michie CA, Davis R, Witkover AD, Rossor M, Nowak MA, Rudge P, Matutes E, Bangham CRM, Weber JN (1999). Effect of lamivudine on human T-cell leukemia virus type 1 (HTLV-1) DNA copy number, T-cell phenotype, and anti-tax cytotoxic T-cell frequency in patients with HTLV-1-associated myelopathy. Journal of Virology.

[B357] Taylor GP, Goon P, Furukawa Y, Green H, Barfield A, Mosley A, Nose H, Babiker A, Rudge P, Usuku K, Osame M, Bangham CR, Weber JN (2006). Zidovudine plus lamivudine in Human T-Lymphotropic Virus type-I-associated myelopathy: a randomised trial. Retrovirology.

[B358] Bazarbachi A, Ghez D, Lepelletier Y, Nasr R, de The H, El Sabban ME, Hermine O (2004). New therapeutic approaches for adult T-cell leukaemia. Lancet Oncol.

